# Supplementary material for: Effects of hydrogeochemistry on the microbial ecology of terrestrial hot springs
Source: Microbiol Spectr. 2023 Sep 27;11(5):e00249-23. doi: 10.1128/spectrum.00249-23 (PMC10581198; doi:10.1128/spectrum.00249-23)
Supplement: Figures S1 to S11, Tables S1 to S4, Text S1 — The file contains all the supplementary material of the manuscript [file spectrum.00249-23-s0001.docx]

# Supplementary Material

## Supplementary Figures


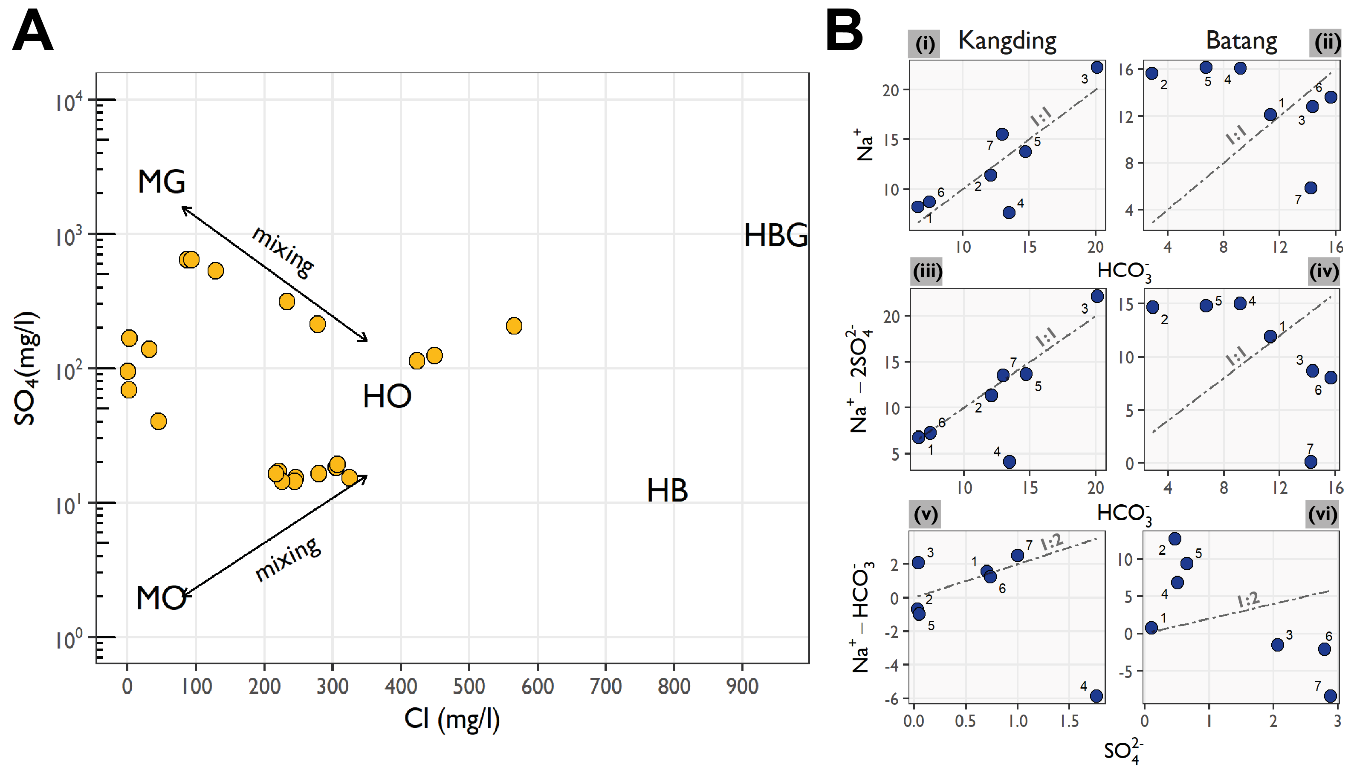


Supplementary Figure 1 A. SO_4_^2-^ concentrations of the YPVF samples plotted against its Cl^-^ concentrations. HO: hydrothermal only, HB: hydrothermal with subsurface boiling, MO: meteoric only, MG: meteoric water with hot gas discharges, HBG: hydrothermal with subsurface boiling hot gas discharge. Proposed by Nordstrom et al. (2009). B. Molar concentrations of Na^+^, SO_4_^2-^ and HCO_3_^-^ in samples from Batang and Kangding geothermal fields. Dashed lines show the stoichiometric ratios of dissolution of albite in presence of CO_2_ (i-ii) and dissolution of albite in presence of CO_2_ and H_2_S (iii-vi).


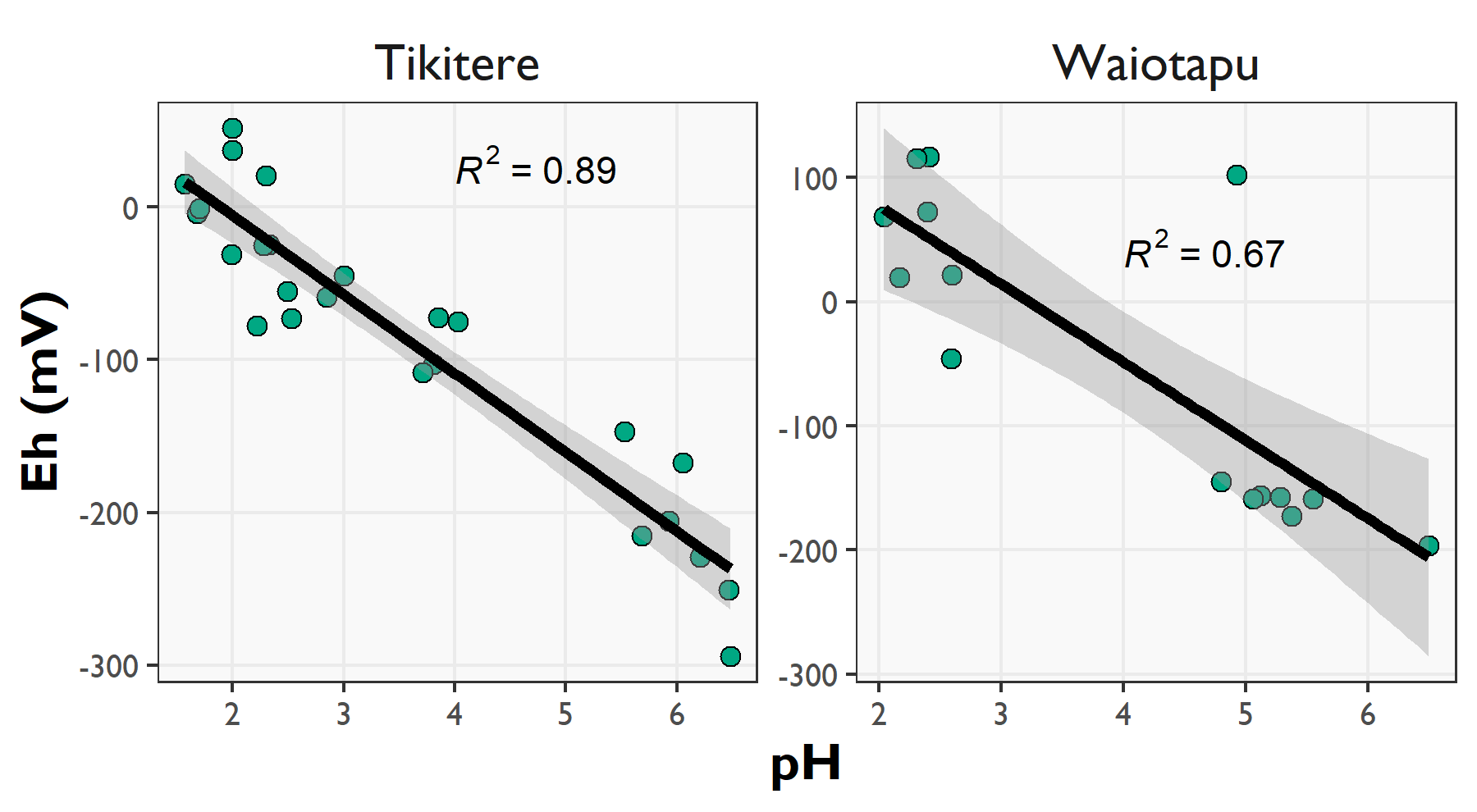


Supplementary Figure 2 Eh vs pH values of samples in Tikitere and Waiotapu geothermal field. Eh values were obtained from One Thousand Spring project website.


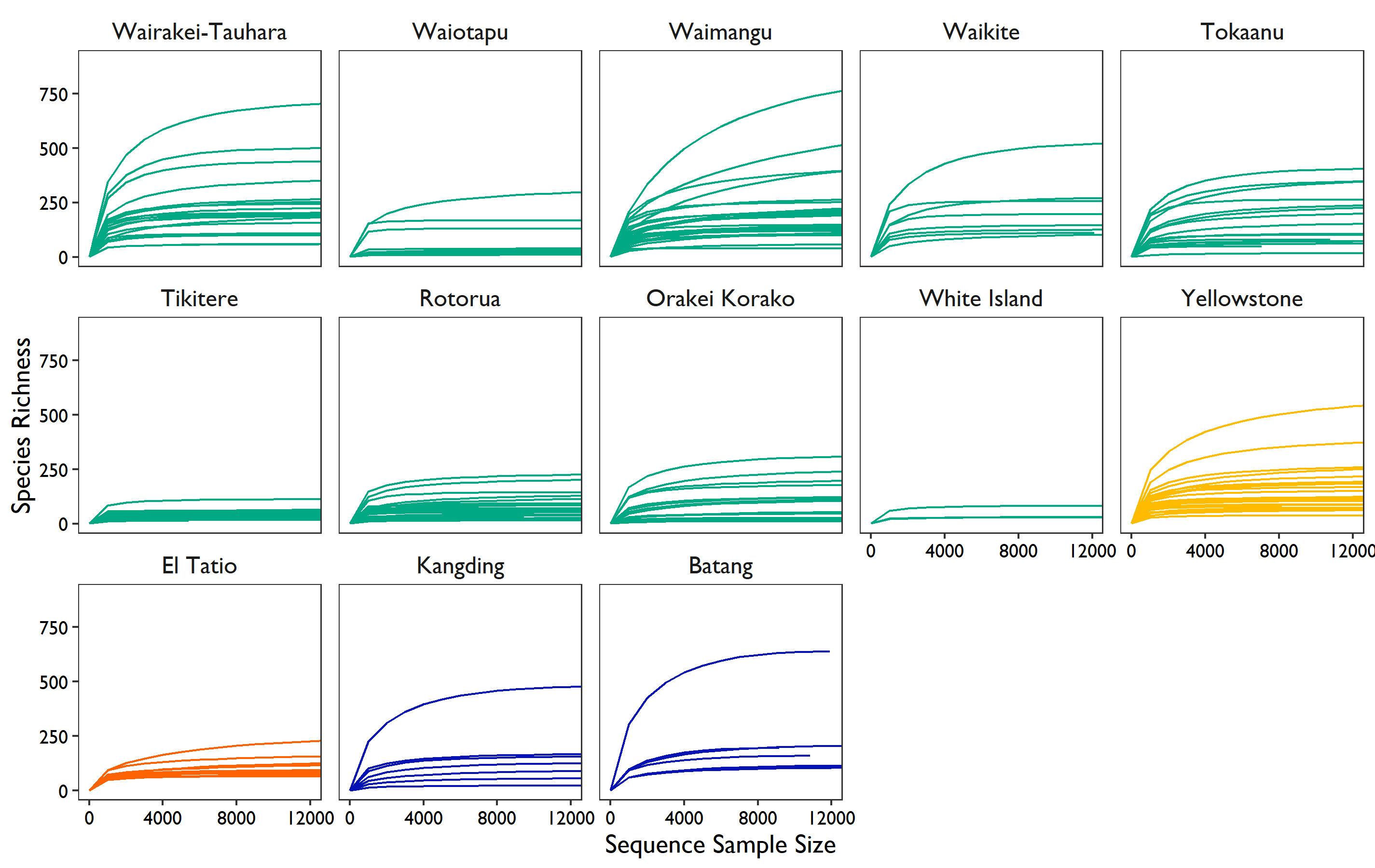


Supplementary Figure 3 Rarefaction curves of the samples by geothermal field.


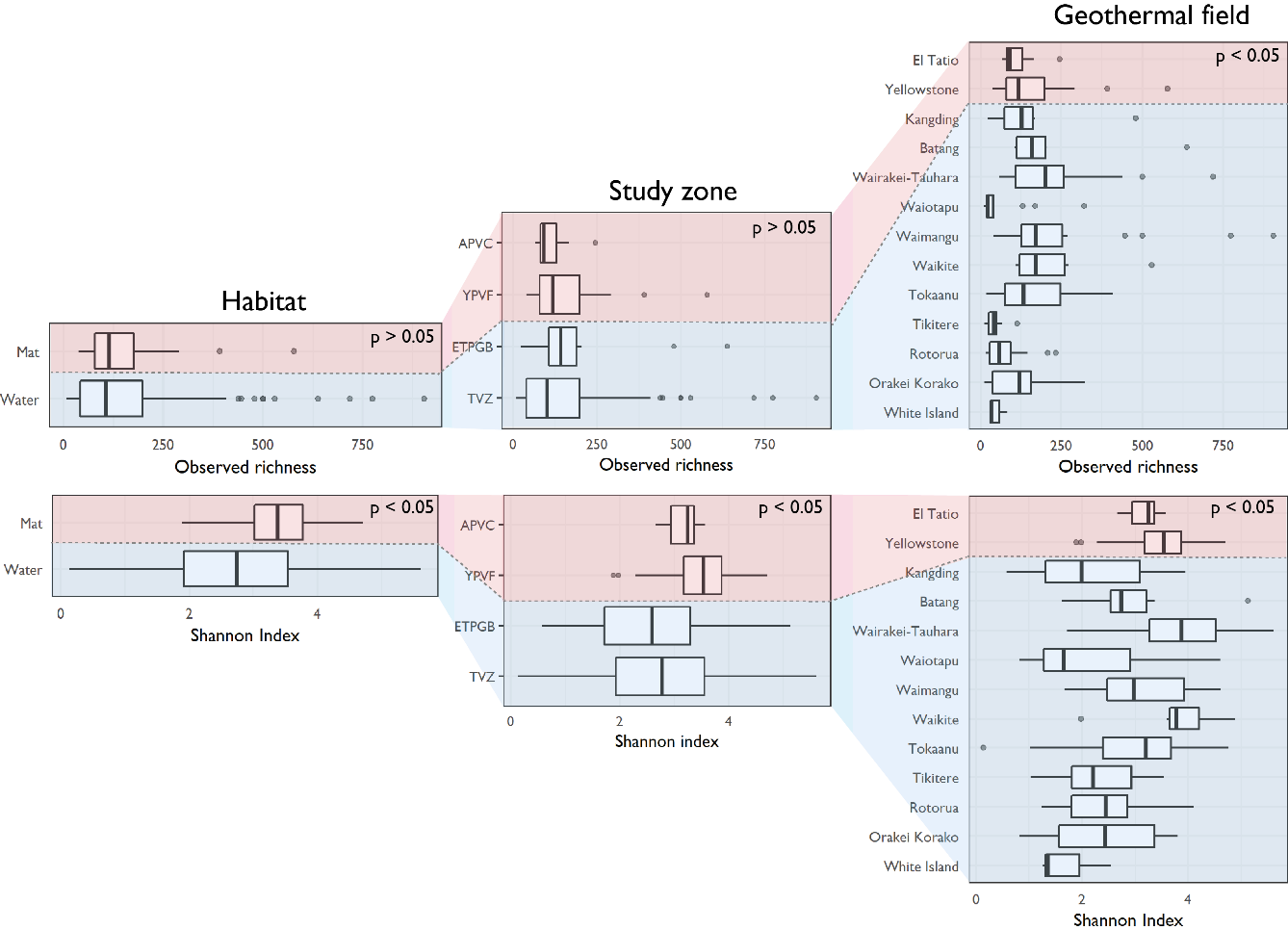


Supplementary Figure 4 Observed richness and Shannon indexes according to the habitat (water or microbial mat), geothermal area and geothermal field (APVC: Altiplano-Puna Volcanic Complex; YPVF: Yellowstone Plateau Volcanic Field; ETPGB: Eastern Tibetan Plateau Geothermal Belt; TVZ: Taupo Volcanic Zone).


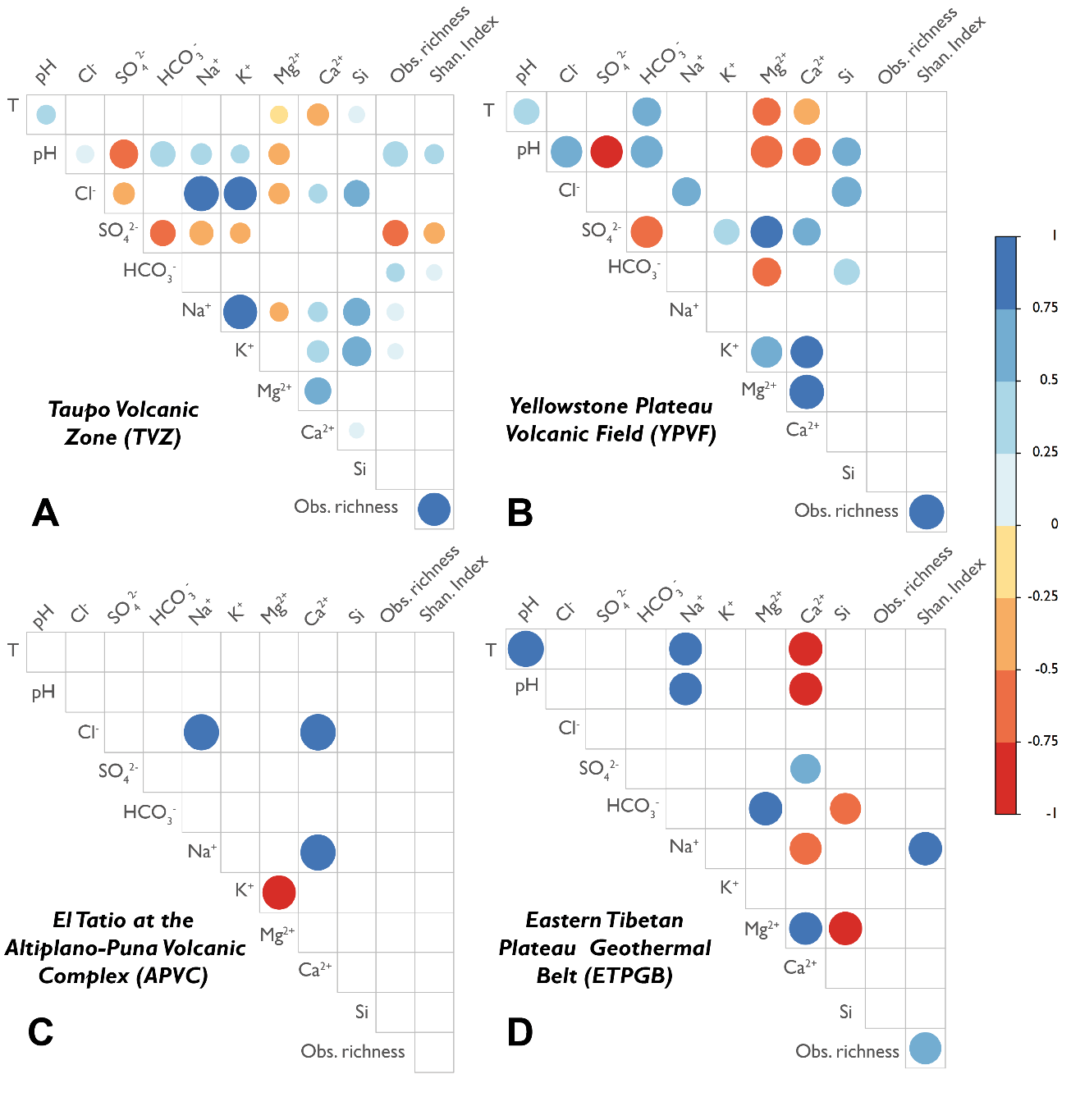


Supplementary Figure 5 Significant Spearman’s correlations between environmental parameters, observed richness and Shannon indexes at each study zone. T: temperature, EC: electrical conductivity, Obs.: observed, Shan: Shannon.


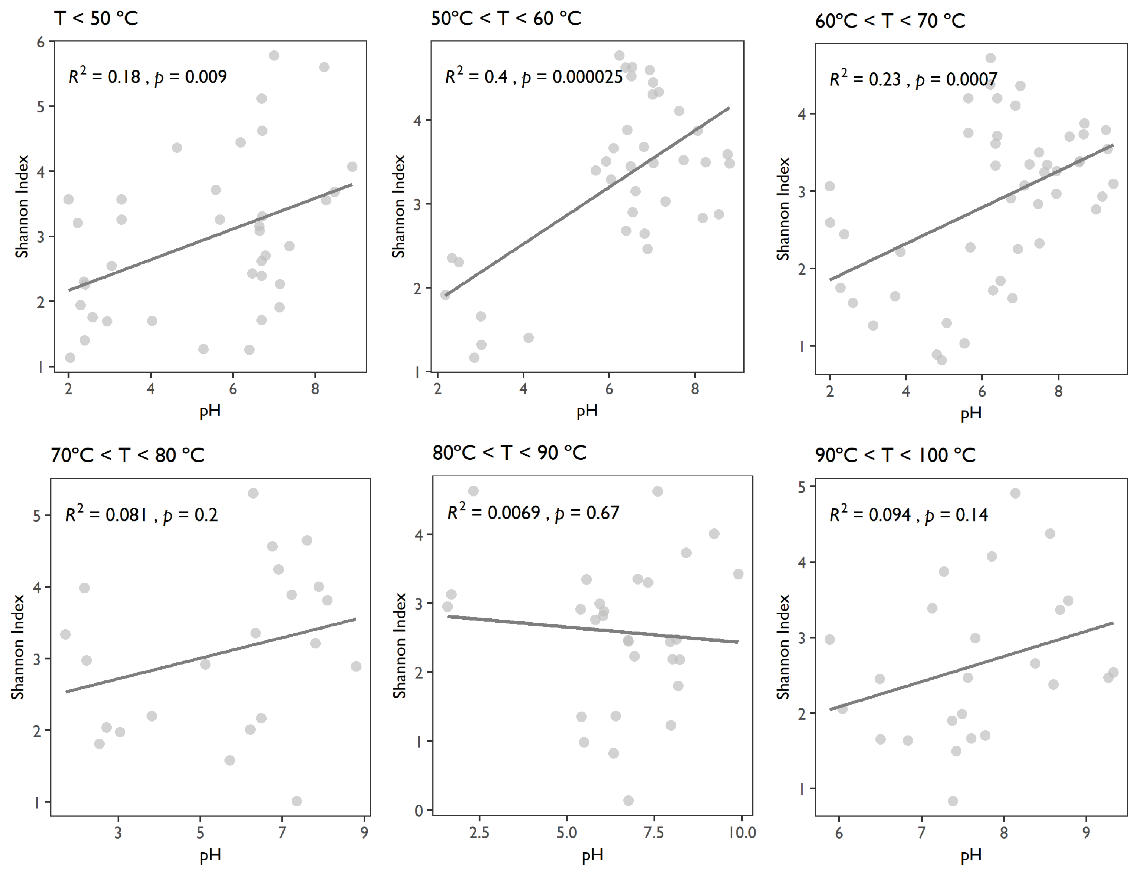


Supplementary Figure 6 Shannon diversity indexes plotted against pH. Samples were divided into six groups according water temperature.


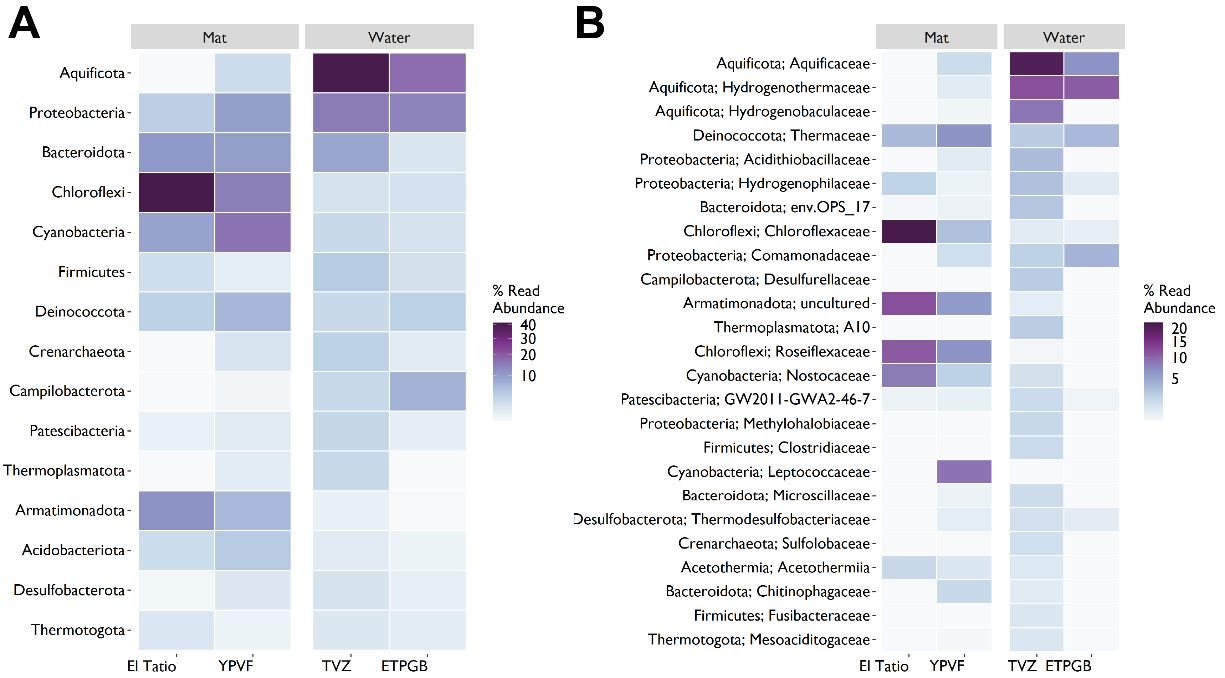


Supplementary Figure 7 Relative abundance of the microbial communities by study zone. A. Abundance of the 15 most abundant phyla. B. Abundance of the 30 most abundant families.


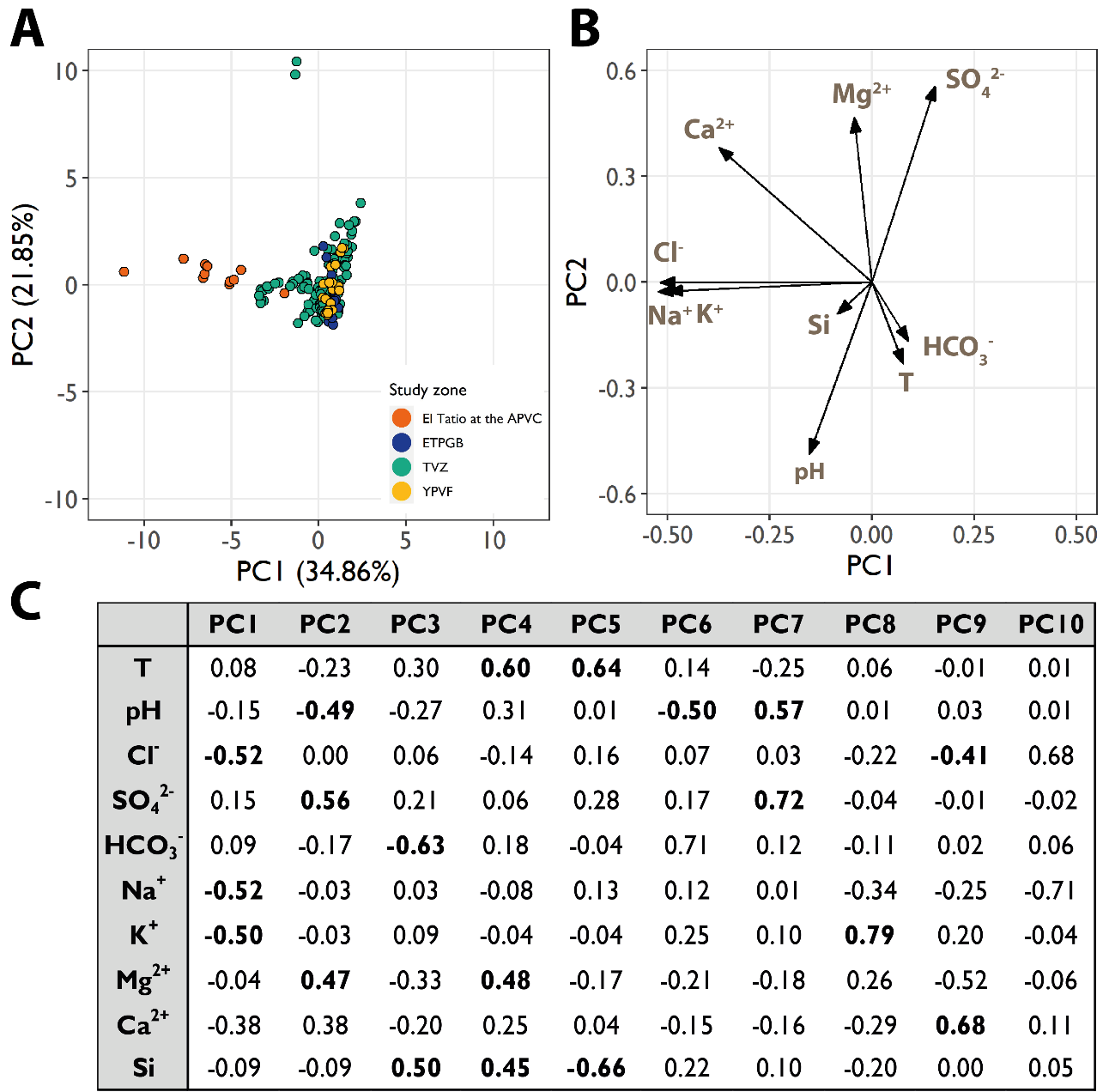


Supplementary Figure 8 Principal component analysis (PCA) of the hydrochemical variables for all the analyzed hot springs. A. Distribution of the analyzed samples in PC1 and PC2 B. Resulted eigenvectors in PC1 and PC2 C.Table with the resulting eigenvalues.


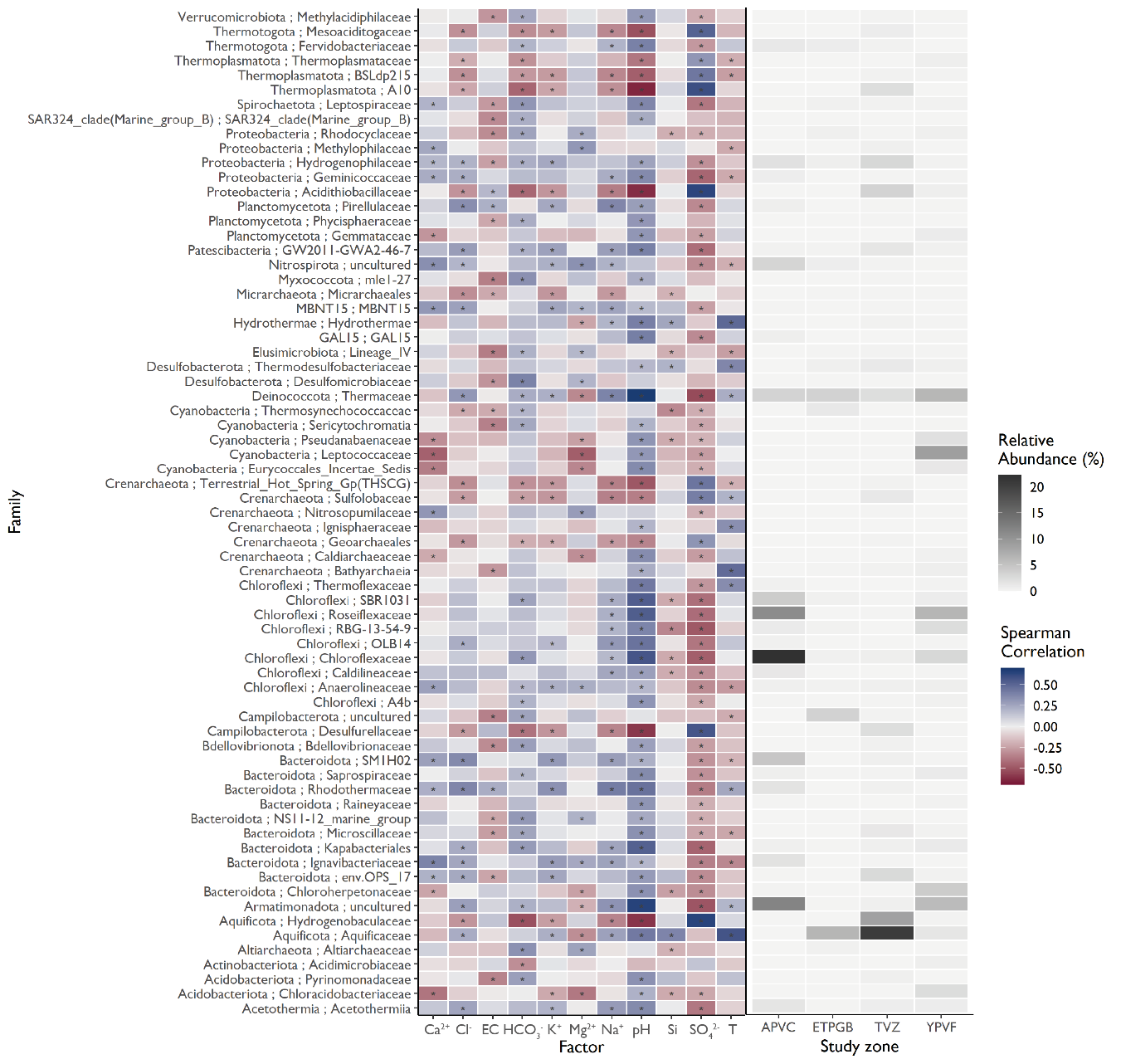


Supplementary Figure 9 Highest Spearman correlations (ρ > 0.3; p > 0.01) between family abundance and hydrochemical variables. Sequences were previously filtered by relative abundance (<0.1%).


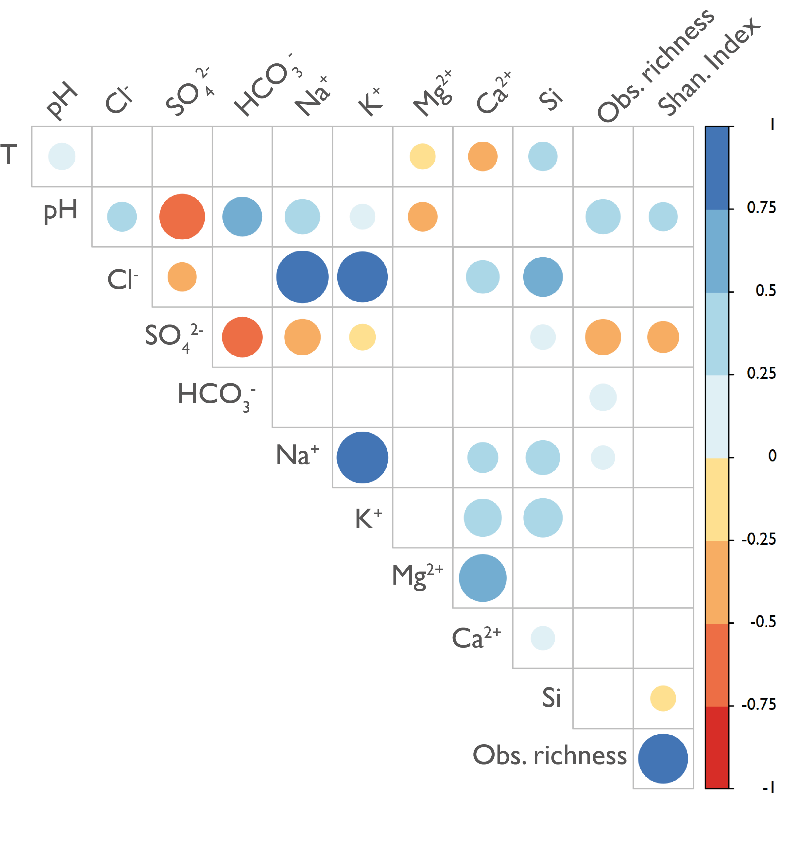


Supplementary Figure 10 Significant Spearman’s correlations between environmental parameters, observed richness and Shannon indexes from all the analyzed hot springs.


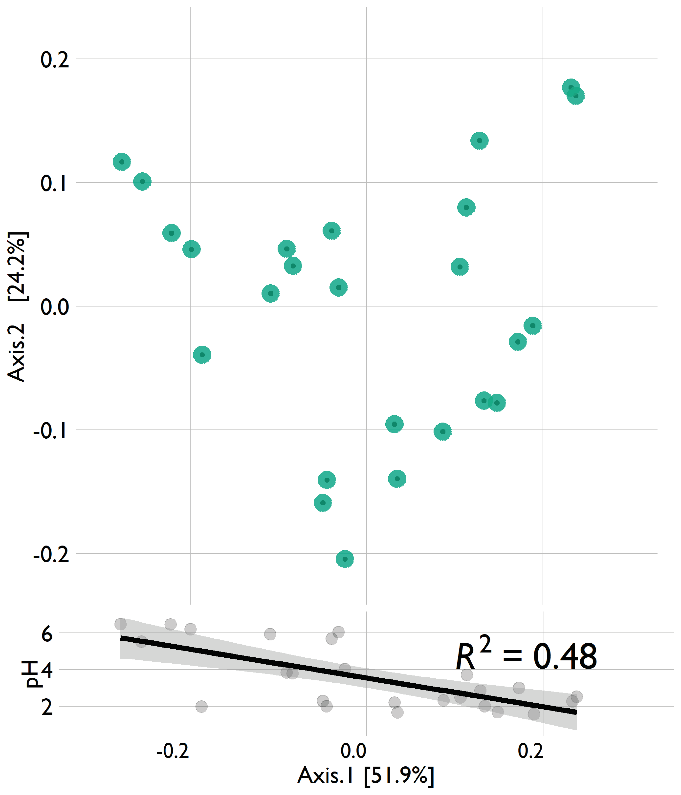


Supplementary Figure 11 Axis 1 and 2 resulted from the MDS analysis performed on the weighted Unifrac distances between thermal microbial communities in Tikitere. Correlation of Axis 1 with pH is also shown

## Supplementary Tables

Supplementary Table 1 Physicochemical parameters and concentration of major elements of the analyzed hot springs.

| **SRA number** | **Area** | **Geothermal field** | **N°** | **Latitude** | **Longitude** | **T (°C)** | **pH** | **EC (μS/cm)** | **Cl^-^ (mg/l)** | **SO_4_^2-^  (mg/l)** | **HCO_3_^-^  (mg/l)** | **Na^+^  (mg/l)** | **K^+^  (mg/l)** | **Mg^2+^  (mg/l)** | **Ca^2+^  (mg/l)** | **Si  (mg/l)** |
| --- | --- | --- | --- | --- | --- | --- | --- | --- | --- | --- | --- | --- | --- | --- | --- | --- |
| SRR18711309 | APVF | El Tatio | 1 | -22.346595 | -68.00834 | 60.7 | 6.75 | 12350 | 3929.00 | 38.01 | 136.00 | 2460.00 | 118.00 | 11.03 | 163.40 | 99.50 |
| SRR18711297 | APVF | El Tatio | 2 | -22.336902 | -68.018595 | 62.0 | 8.54 | 11700 | 3796.00 | 35.49 | 95.00 | 2250.00 | 229.00 | 5.93 | 141.90 | 70.99 |
| SRR18711284 | APVF | El Tatio | 3 | -22.337869 | -68.01719 | 61.0 | 7.94 | 11500 | 3808.00 | 34.87 | 107.00 | 2260.00 | 230.00 | 5.22 | 141.00 | 66.32 |
| SRR18711281 | APVF | El Tatio | 4 | -22.356122 | -68.022753 | 50.0 | 9.27 | 7700 | 1679.00 | 66.38 | 237.00 | 1130.00 | 96.00 | 11.34 | 77.50 | 126.66 |
| SRR18711307 | APVF | El Tatio | 5 | -22.337869 | -68.01719 | 62.8 | 7.63 | 14600 | 4716.00 | 35.10 | 74.00 | 2890.00 | 286.00 | 4.94 | 169.40 | 86.00 |
| SRR18711304 | APVF | El Tatio | 6 | -22.34558 | -68.012261 | 65.0 | 7.23 | 16500 | 5394.00 | 46.79 | 42.00 | 3270.00 | 149.00 | 6.77 | 238.40 | 109.79 |
| SRR18711301 | APVF | El Tatio | 7 | -22.337409 | -68.017377 | 55.0 | 7.30 | 14500 | 4706.00 | 35.44 | 91.00 | 2790.00 | 287.00 | 4.91 | 168.50 | 79.87 |
| SRR18711299 | APVF | El Tatio | 8 | -22.336949 | -68.0144 | 60.0 | 7.45 | 11650 | 3537.00 | 35.25 | 96.00 | 2270.00 | 212.00 | 5.03 | 140.30 | 96.37 |
| SRR18711295 | APVF | El Tatio | 9 | -22.333578 | -68.012773 | 50.0 | 8.24 | 21000 | 7061.00 | 36.30 | 14.00 | 4260.00 | 508.00 | 1.72 | 239.60 | 113.01 |
| SRR18711292 | APVF | El Tatio | 10 | -22.347724 | -68.011304 | 60.0 | 7.12 | 14400 | 5757.00 | 51.66 | 140.00 | 4580.00 | 152.00 | 10.48 | 258.80 | 84.08 |
| SRR18711289 | APVF | El Tatio | 11 | -22.350396 | -68.007983 | 62.7 | 7.48 | 15000 | 5286.00 | 46.17 | 45.00 | 3210.00 | 150.00 | 4.98 | 230.60 | 95.34 |
| SRR8420032 | YPVF | Yellowstone | 1 | 44.569662 | -110.865018 | 55.0 | 8.24 | 1439 | 220.87 | 17.10 | 263.13 | 280.71 | 15.64 | 0.00 | 0.36 | 93.23 |
| SRR8420033 | YPVF | Yellowstone | 8 | 44.687666 | -110.727923 | 44.2 | 8.26 | 2481 | 449.54 | 123.92 | 5.74 | 392.44 | 28.54 | 0.24 | 4.81 | 112.60 |
| SRR8420034 | YPVF | Yellowstone | 9 | 44.687602 | -110.727684 | 59.9 | 7.62 | 3254 | 424.02 | 113.35 | 191.95 | 364.39 | 27.76 | 0.24 | 4.81 | 111.20 |
| SRR8420035 | YPVF | Yellowstone | 10 | 44.532526 | -110.797769 | 71.0 | 8.80 | 2910 | 246.04 | 15.37 | 0.02 | 346.69 | 21.50 | 0.00 | 0.52 | 130.01 |
| SRR8420036 | YPVF | Yellowstone | 11 | 44.610054 | -110.438726 | 63.7 | 6.21 | 1100 | 45.38 | 40.35 | 0.02 | 78.63 | 28.93 | 15.07 | 30.46 | 55.88 |
| SRR8420037 | YPVF | Yellowstone | 12 | 44.61019 | -110.438914 | 58.2 | 5.68 | 2539 | 278.31 | 213.25 | 9.61 | 296.34 | 37.53 | 0.97 | 6.41 | 78.34 |
| SRR8420038 | YPVF | Yellowstone | 13 | 44.610107 | -110.43865 | 31.5 | 4.64 | 556 | 31.91 | 138.33 | 5.74 | 63.68 | 17.59 | 6.81 | 15.63 | 44.65 |
| SRR8420039 | YPVF | Yellowstone | 14 | 44.558769 | -110.843889 | 68.5 | 8.68 | 2944 | 280.08 | 16.33 | 119.56 | 342.32 | 11.73 | 0.00 | 1.16 | 59.81 |
| SRR8420040 | YPVF | Yellowstone | 15 | 44.532499 | -110.797668 | 69.4 | 8.56 | 2751 | 244.27 | 14.41 | 127.41 | 337.95 | 20.72 | 0.00 | 0.60 | 111.20 |
| SRR8420041 | YPVF | Yellowstone | 16 | 44.5684 | -110.863623 | 51.4 | 8.05 | 2748 | 225.84 | 14.41 | 242.60 | 316.34 | 13.68 | 0.00 | 0.56 | 63.46 |
| SRR8420042 | YPVF | Yellowstone | 2 | 44.517642 | -110.806477 | 67.9 | 5.63 | 460 | 2.48 | 167.14 | 205.99 | 76.79 | 15.64 | 0.02 | 0.60 | 69.36 |
| SRR8420043 | YPVF | Yellowstone | 3 | 44.700569 | -110.765528 | 69.5 | 6.39 | 1925 | 232.93 | 314.12 | 63.01 | 383.01 | 27.37 | 0.02 | 3.25 | 71.60 |
| SRR8420044 | YPVF | Yellowstone | 4 | 44.729157 | -110.712676 | 38.4 | 3.29 | 2470 | 0.35 | 95.10 | 14.84 | 373.36 | 46.92 | 0.10 | 4.37 | 51.95 |
| SRR8420045 | YPVF | Yellowstone | 17 | 44.569653 | -110.865005 | 68.4 | 7.95 | 2969 | 216.97 | 16.33 | 129.75 | 287.83 | 14.08 | 0.00 | 0.44 | 101.09 |
| SRR8420046 | YPVF | Yellowstone | 5 | 44.729157 | -110.712676 | 38.4 | 3.29 | 2470 | 0.35 | 95.10 | 266.93 | 373.36 | 46.92 | 0.10 | 4.37 | 51.95 |
| SRR8420047 | YPVF | Yellowstone | 6 | 44.699573 | -110.767223 | 40.5 | 7.13 | 2310 | 565.48 | 206.53 | 257.68 | 404.85 | 35.19 | 0.07 | 5.17 | 61.50 |
| SRR8420048 | YPVF | Yellowstone | 7 | 44.517642 | -110.806477 | 67.9 | 5.63 | 460 | 2.48 | 167.14 | 319.63 | 76.79 | 15.64 | 0.02 | 0.60 | 69.36 |
| SRR8420049 | YPVF | Yellowstone | 18 | 44.516526 | -110.806695 | 65.1 | 6.20 | 510 | 1.77 | 69.16 | 38.53 | 49.20 | 10.95 | 0.00 | 0.40 | 38.47 |
| SRR8420050 | YPVF | Yellowstone | 19 | 44.520461 | -110.812571 | 62.4 | 9.44 | 3756 | 324.75 | 15.37 | 137.99 | 371.75 | 17.20 | 0.00 | 0.40 | 117.66 |
| SRR8420052 | YPVF | Yellowstone | 20 | 44.700081 | -110.764873 | 52.8 | 2.17 | 4573 | 87.21 | 640.72 | 0.03 | 82.76 | 21.11 | 0.39 | 3.53 | 57.00 |
| SRR8420053 | YPVF | Yellowstone | 21 | 44.700009 | -110.764874 | 49.1 | 2.38 | 3804 | 128.69 | 529.29 | 0.08 | 109.89 | 23.85 | 0.49 | 4.33 | 71.04 |
| SRR8420054 | YPVF | Yellowstone | 22 | 44.520407 | -110.812558 | 62.3 | 9.29 | 3578 | 304.90 | 18.25 | 164.71 | 363.01 | 15.64 | 0.00 | 0.40 | 96.31 |
| SRR8420055 | YPVF | Yellowstone | 23 | 44.5199 | -110.8115 | 62.3 | 9.24 | 3584 | 305.25 | 18.25 | 174.04 | 361.17 | 15.25 | 0.00 | 0.40 | 69.64 |
| SRR8420056 | YPVF | Yellowstone | 24 | 44.52091 | -110.812154 | 68.4 | 9.14 | 3944 | 307.38 | 19.21 | 177.29 | 363.47 | 15.64 | 0.00 | 0.40 | 88.17 |
| SRR8420057 | YPVF | Yellowstone | 25 | 44.700251 | -110.764254 | 47.2 | 2.22 | 4133 | 93.95 | 644.56 | 0.00 | 94.03 | 22.68 | 0.39 | 3.61 | 76.94 |
| SRR10580885 | ETPGB | Batang | 1 | 30.26 | 99.46 | 65.4 | 7.10 | 1200 | 16.80 | 5.10 | 692.50 | 278.30 | 21.40 | 1.70 | 4.10 | 27.58 |
| SRR10580888 | ETPGB | Batang | 2 | 30.4 | 99.4 | 88.2 | 9.90 | 1430 | 50.90 | 22.60 | 176.50 | 359.00 | 29.30 | 0.00 | 0.60 | 70.36 |
| SRR10580889 | ETPGB | Batang | 3 | 30.28 | 99.34 | 64.4 | 7.70 | 1580 | 14.40 | 99.00 | 876.10 | 294.20 | 20.00 | 17.80 | 26.60 | 29.91 |
| SRR10580891 | ETPGB | Batang | 4 | 30.4 | 99.39 | 88.0 | 9.20 | 1530 | 53.20 | 24.60 | 560.80 | 368.80 | 30.10 | 0.10 | 0.50 | 56.30 |
| SRR10580894 | ETPGB | Batang | 5 | 30.4 | 99.38 | 87.8 | 8.40 | 820 | 51.60 | 31.50 | 411.50 | 370.50 | 31.40 | 0.00 | 1.30 | 74.82 |
| SRR10580896 | ETPGB | Batang | 6 | 30.15 | 99.19 | 45.5 | 7.00 | 1940 | 11.20 | 134.10 | 957.70 | 312.50 | 26.30 | 40.40 | 49.80 | 27.07 |
| SRR10580898 | ETPGB | Batang | 7 | 29.96 | 99.08 | 43.3 | 6.70 | 1490 | 14.20 | 138.50 | 868.40 | 134.50 | 34.00 | 68.40 | 116.10 | 25.60 |
| SRR10580884 | ETPGB | Kangding | 1 | 30.18 | 101.87 | 63.6 | 7.50 | 880 | 53.00 | 33.80 | 403.00 | 187.90 | 14.80 | 1.40 | 22.00 | 56.84 |
| SRR10580886 | ETPGB | Kangding | 2 | 30.28 | 101.94 | 37.4 | 6.70 | 1240 | 53.50 | 1.60 | 738.40 | 262.00 | 18.90 | 5.10 | 24.50 | 40.09 |
| SRR10580890 | ETPGB | Kangding | 3 | 29.98 | 101.96 | 72.2 | 7.60 | 2680 | 142.30 | 2.10 | 1228.10 | 510.60 | 53.80 | 32.80 | 17.90 | 34.98 |
| SRR10580892 | ETPGB | Kangding | 4 | 30.12 | 101.94 | 40.4 | 6.40 | 2320 | 57.30 | 84.70 | 822.50 | 174.80 | 28.80 | 49.50 | 94.50 | 14.06 |
| SRR10580895 | ETPGB | Kangding | 5 | 30.26 | 101.87 | 60.8 | 7.00 | 1750 | 75.60 | 2.40 | 899.40 | 316.00 | 30.30 | 15.10 | 20.50 | 40.66 |
| SRR10580897 | ETPGB | Kangding | 6 | 30.18 | 101.87 | 44.8 | 6.70 | 960 | 58.60 | 35.40 | 455.30 | 200.20 | 15.90 | 2.00 | 33.70 | 60.22 |
| SRR10580899 | ETPGB | Kangding | 7 | 29.95 | 101.96 | 79.5 | 7.80 | 2420 | 126.90 | 48.00 | 791.90 | 356.20 | 41.70 | 18.10 | 24.40 | 54.87 |
| ERR2240344 | TVZ | Orakei Korako | 1 | -38.4736 | 176.1467 | 50.6 | 8.80 | 1817 | 345.00 | 97.00 | 219.00 | 291.00 | 38.00 | 0.26 | 5.30 | 120.00 |
| ERR2240345 | TVZ | Orakei Korako | 2 | -38.4735 | 176.1468 | 75.8 | 8.09 | 1693 | 320.00 | 97.00 | 225.00 | 271.00 | 35.00 | 0.30 | 5.40 | 109.00 |
| ERR2240346 | TVZ | Orakei Korako | 3 | -38.4734 | 176.1469 | 82.3 | 7.94 | 1666 | 313.00 | 96.00 | 216.00 | 269.00 | 36.00 | 0.47 | 5.90 | 109.00 |
| ERR2240347 | TVZ | Orakei Korako | 4 | -38.4731 | 176.1475 | 99.0 | 7.42 | 1585 | 305.00 | 121.00 | 134.00 | 254.00 | 36.00 | 0.34 | 8.30 | 114.00 |
| ERR2240348 | TVZ | Orakei Korako | 5 | -38.4732 | 176.1475 | 93.9 | 8.38 | 1637 | 319.00 | 101.00 | 168.00 | 263.00 | 36.00 | 0.34 | 6.80 | 120.00 |
| ERR2240349 | TVZ | Orakei Korako | 6 | -38.4732 | 176.1482 | 97.2 | 6.83 | 1731 | 328.00 | 114.00 | 223.00 | 297.00 | 39.00 | 0.34 | 8.70 | 115.00 |
| ERR2241038 | TVZ | Orakei Korako | 7 | -38.4733 | 176.1471 | 93.6 | 7.13 | 2028 | 283.00 | 90.00 | 230.00 | 340.00 | 43.00 | 1.50 | 7.00 | 124.00 |
| ERR2241039 | TVZ | Orakei Korako | 8 | -38.4735 | 176.1483 | 97.8 | 7.38 | 2270 | 308.00 | 99.00 | 265.00 | 365.00 | 51.00 | 1.10 | 7.50 | 144.00 |
| ERR2241040 | TVZ | Orakei Korako | 9 | -38.4736 | 176.1483 | 85.3 | 7.02 | 2154 | 278.00 | 149.00 | 192.00 | 327.00 | 48.00 | 0.99 | 8.40 | 140.00 |
| ERR2241041 | TVZ | Orakei Korako | 10 | -38.4735 | 176.1482 | 58.3 | 7.73 | 2375 | 318.00 | 104.00 | 264.00 | 356.00 | 52.00 | 0.98 | 10.60 | 133.00 |
| ERR2241044 | TVZ | Orakei Korako | 11 | -38.4735 | 176.1468 | 89.3 | 6.33 | 2165 | 287.00 | 90.00 | 294.00 | 333.00 | 40.00 | 0.84 | 6.60 | 138.00 |
| ERR2241045 | TVZ | Orakei Korako | 12 | -38.4737 | 176.1468 | 83.8 | 5.48 | 1908 | 279.00 | 152.00 | 67.00 | 276.00 | 33.00 | 1.10 | 7.60 | 127.00 |
| ERR2241046 | TVZ | Orakei Korako | 13 | -38.4742 | 176.1485 | 85.5 | 6.74 | 2100 | 321.00 | 120.00 | 206.00 | 457.00 | 52.00 | 3.80 | 7.40 | 173.00 |
| ERR2241047 | TVZ | Orakei Korako | 14 | -38.474 | 176.1485 | 96.0 | 7.77 | 2116 | 322.00 | 116.00 | 191.00 | 434.00 | 50.00 | 3.30 | 9.10 | 161.00 |
| ERR2241048 | TVZ | Orakei Korako | 15 | -38.4738 | 176.1471 | 95.0 | 7.37 | 1976 | 319.00 | 76.00 | 288.00 | 357.00 | 46.00 | 0.95 | 6.60 | 158.00 |
| ERR2240422 | TVZ | Rotorua | 1 | -38.1331 | 176.2454 | 81.3 | 6.03 | 1700 | 289.00 | 117.00 | 154.00 | 260.00 | 16.30 | 0.62 | 12.20 | 122.00 |
| ERR2240473 | TVZ | Rotorua | 2 | -38.1318 | 176.2453 | 87.1 | 6.92 | 1631 | 327.00 | 74.00 | 262.00 | 332.00 | 24.00 | 0.98 | 9.90 | 145.00 |
| ERR2240483 | TVZ | Rotorua | 3 | -38.1318 | 176.2443 | 49.2 | 2.94 | 2158 | 235.00 | 378.00 | 131.00 | 224.00 | 20.00 | 2.30 | 30.00 | 114.00 |
| ERR2240508 | TVZ | Rotorua | 4 | -38.1297 | 176.2445 | 54.2 | 8.17 | 1396 | 239.00 | 60.00 | 205.00 | 239.00 | 14.50 | 0.56 | 6.70 | 101.00 |
| ERR2240516 | TVZ | Rotorua | 5 | -38.1657 | 176.2505 | 56.8 | 8.55 | 1824 | 482.00 | 109.00 | 85.00 | 351.00 | 43.00 | 0.27 | 5.90 | 152.00 |
| ERR2240588 | TVZ | Rotorua | 6 | -38.1297 | 176.2443 | 46.8 | 7.37 | 1233 | 248.00 | 69.00 | 222.00 | 265.00 | 18.90 | 0.39 | 7.10 | 109.00 |
| ERR2240589 | TVZ | Rotorua | 7 | -38.1298 | 176.2438 | 53.2 | 6.89 | 1570 | 324.00 | 84.00 | 273.00 | 314.00 | 26.00 | 0.36 | 7.30 | 118.00 |
| ERR2240629 | TVZ | Rotorua | 8 | -38.164 | 176.2522 | 93.8 | 9.27 | 2252 | 563.00 | 52.00 | 84.00 | 418.00 | 29.00 | 0.39 | 6.40 | 105.00 |
| ERR2240637 | TVZ | Rotorua | 9 | -38.1309 | 176.2441 | 59.5 | 4.11 | 1359 | 208.00 | 158.00 | 44.00 | 172.00 | 19.70 | 0.82 | 10.10 | 136.00 |
| ERR2240654 | TVZ | Rotorua | 10 | -38.1641 | 176.253 | 73.2 | 2.71 | 4583 | 373.00 | 515.00 | 24.00 | 285.00 | 30.00 | 0.36 | 5.40 | 153.00 |
| ERR2240662 | TVZ | Rotorua | 11 | -38.1642 | 176.2528 | 72.0 | 3.04 | 3560 | 425.00 | 370.00 | 34.00 | 320.00 | 36.00 | 0.28 | 5.60 | 141.00 |
| ERR2240672 | TVZ | Rotorua | 12 | -38.1621 | 176.2555 | 79.6 | 6.35 | 2560 | 490.00 | 184.00 | 65.00 | 385.00 | 36.00 | 0.45 | 7.60 | 154.00 |
| ERR2240680 | TVZ | Rotorua | 13 | -38.1643 | 176.2528 | 55.9 | 3.01 | 3468 | 464.00 | 418.00 | 48.00 | 378.00 | 44.00 | 0.85 | 5.60 | 144.00 |
| ERR2240707 | TVZ | Rotorua | 14 | -38.1621 | 176.2565 | 67.5 | 7.46 | 2811 | 499.00 | 197.00 | 39.00 | 404.00 | 37.00 | 0.39 | 6.50 | 130.00 |
| ERR2240712 | TVZ | Rotorua | 15 | -38.1619 | 176.2565 | 94.1 | 6.04 | 2658 | 482.00 | 193.00 | 39.00 | 392.00 | 37.00 | 0.69 | 7.80 | 133.00 |
| ERR2240716 | TVZ | Rotorua | 16 | -38.1612 | 176.2579 | 58.9 | 7.02 | 2363 | 694.00 | 185.00 | 40.00 | 527.00 | 39.00 | 0.66 | 11.90 | 97.00 |
| ERR2240728 | TVZ | Rotorua | 17 | -38.1611 | 176.258 | 61.5 | 6.87 | 2261 | 691.00 | 146.00 | 61.00 | 509.00 | 34.00 | 0.24 | 8.70 | 94.00 |
| ERR2240756 | TVZ | Rotorua | 18 | -38.1619 | 176.2592 | 79.3 | 5.72 | 2495 | 480.00 | 229.00 | 24.00 | 410.00 | 49.00 | 0.35 | 7.40 | 124.00 |
| ERR2240786 | TVZ | Rotorua | 19 | -38.1625 | 176.2583 | 86.5 | 8.18 | 2564 | 549.00 | 181.00 | 86.00 | 448.00 | 52.00 | 0.73 | 10.80 | 141.00 |
| ERR2240797 | TVZ | Rotorua | 20 | -38.1628 | 176.2545 | 86.8 | 6.39 | 2410 | 510.00 | 156.00 | 83.00 | 422.00 | 34.00 | 0.32 | 8.10 | 92.00 |
| ERR2240800 | TVZ | Rotorua | 21 | -38.1627 | 176.2581 | 88.0 | 7.97 | 2330 | 571.00 | 122.00 | 147.00 | 486.00 | 45.00 | 0.26 | 8.10 | 116.00 |
| ERR2240809 | TVZ | Rotorua | 22 | -38.1628 | 176.2582 | 92.1 | 8.60 | 2153 | 543.00 | 158.00 | 57.00 | 429.00 | 44.00 | 0.25 | 6.40 | 124.00 |
| ERR2240825 | TVZ | Rotorua | 23 | -38.1629 | 176.2548 | 58.9 | 6.54 | 2666 | 489.00 | 179.00 | 85.00 | 404.00 | 54.00 | 0.24 | 7.90 | 85.00 |
| ERR2240865 | TVZ | Rotorua | 24 | -38.163 | 176.2544 | 66.9 | 2.37 | 3216 | 330.00 | 428.00 | 51.00 | 270.00 | 38.00 | 0.44 | 6.40 | 119.00 |
| ERR2240874 | TVZ | Rotorua | 25 | -38.1638 | 176.2542 | 98.5 | 8.68 | 2492 | 607.00 | 111.00 | 186.00 | 512.00 | 70.00 | 0.13 | 5.00 | 186.00 |
| ERR2240258 | TVZ | Tikitere | 1 | -38.0611 | 176.3593 | 46.6 | 4.03 | 1120 | 2.20 | 403.00 | 213.00 | 11.70 | 5.10 | 2.30 | 12.50 | 80.00 |
| ERR2240260 | TVZ | Tikitere | 2 | -38.061 | 176.3592 | 57.0 | 3.00 | 1359 | 2.10 | 406.00 | 88.00 | 11.70 | 6.30 | 1.80 | 10.70 | 120.00 |
| ERR2240270 | TVZ | Tikitere | 3 | -38.0608 | 176.3587 | 88.4 | 1.57 | 12540 | 2.80 | 2418.00 | 33.00 | 10.70 | 6.50 | 1.80 | 10.30 | 107.00 |
| ERR2240271 | TVZ | Tikitere | 4 | -38.0608 | 176.3586 | 82.3 | 1.68 | 9265 | 2.60 | 1779.00 | 40.00 | 14.00 | 8.80 | 1.70 | 11.80 | 110.00 |
| ERR2240272 | TVZ | Tikitere | 5 | -38.0607 | 176.3587 | 73.4 | 1.70 | 8799 | 2.50 | 1708.00 | 43.00 | 13.40 | 9.10 | 1.70 | 9.90 | 113.00 |
| ERR2240273 | TVZ | Tikitere | 6 | -38.0608 | 176.3587 | 57.2 | 2.85 | 1473 | 1.10 | 390.00 | 70.00 | 14.70 | 9.20 | 1.10 | 10.60 | 117.00 |
| ERR2240276 | TVZ | Tikitere | 7 | -38.0609 | 176.3586 | 74.6 | 2.22 | 3265 | 0.90 | 700.00 | 105.00 | 13.00 | 7.40 | 0.83 | 6.90 | 132.00 |
| ERR2240284 | TVZ | Tikitere | 8 | -38.0608 | 176.3582 | 76.6 | 3.81 | 1827 | 1.80 | 695.00 | 117.00 | 14.60 | 8.50 | 5.10 | 18.20 | 98.00 |
| ERR2240285 | TVZ | Tikitere | 9 | -38.0607 | 176.3582 | 64.3 | 5.68 | 2182 | 2.20 | 819.00 | 94.00 | 10.10 | 4.10 | 2.00 | 7.60 | 75.00 |
| ERR2240286 | TVZ | Tikitere | 10 | -38.0607 | 176.3583 | 63.1 | 3.71 | 2337 | 2.30 | 904.00 | 51.00 | 7.80 | 5.90 | 0.77 | 6.00 | 94.00 |
| ERR2240290 | TVZ | Tikitere | 11 | -38.0606 | 176.3586 | 64.2 | 3.85 | 1287 | 1.60 | 462.00 | 38.00 | 9.60 | 5.30 | 1.30 | 6.20 | 94.00 |
| ERR2240292 | TVZ | Tikitere | 12 | -38.0612 | 176.3581 | 80.6 | 5.93 | 4794 | 1.10 | 1596.00 | 35.00 | 3.10 | 2.90 | 0.68 | 6.80 | 55.00 |
| ERR2240393 | TVZ | Tikitere | 13 | -38.0597 | 176.3591 | 54.7 | 2.33 | 3935 | 1.80 | 1231.00 | 104.00 | 5.70 | 4.30 | 0.68 | 6.90 | 88.00 |
| ERR2240400 | TVZ | Tikitere | 14 | -38.0593 | 176.359 | 69.0 | 1.99 | 5464 | 8.20 | 1393.00 | 36.00 | 12.90 | 6.40 | 1.30 | 7.00 | 106.00 |
| ERR2240411 | TVZ | Tikitere | 15 | -38.0593 | 176.3591 | 73.0 | 2.53 | 3050 | 0.09 | 834.00 | 48.00 | 9.30 | 9.30 | 1.90 | 10.90 | 100.00 |
| ERR2240414 | TVZ | Tikitere | 16 | -38.0592 | 176.3592 | 54.4 | 2.49 | 2994 | 0.06 | 780.00 | 39.00 | 10.00 | 9.40 | 2.00 | 12.50 | 93.00 |
| ERR2240417 | TVZ | Tikitere | 17 | -38.059 | 176.359 | 63.3 | 2.28 | 3208 | 0.05 | 808.00 | 22.00 | 15.80 | 7.30 | 3.90 | 16.10 | 131.00 |
| ERR2240434 | TVZ | Tikitere | 18 | -38.0589 | 176.3589 | 60.4 | 6.47 | 1059 | 1.80 | 478.00 | 119.00 | 7.70 | 5.00 | 1.10 | 6.90 | 81.00 |
| ERR2240436 | TVZ | Tikitere | 19 | -38.0587 | 176.3594 | 86.8 | 6.05 | 2343 | 3.40 | 985.00 | 28.00 | 8.10 | 6.00 | 2.30 | 15.50 | 56.00 |
| ERR2240439 | TVZ | Tikitere | 20 | -38.0592 | 176.3604 | 78.5 | 6.48 | 489 | 4.60 | 33.00 | 359.00 | 41.00 | 7.20 | 0.35 | 6.80 | 100.00 |
| ERR2240442 | TVZ | Tikitere | 21 | -38.063 | 176.361 | 74.3 | 6.21 | 236 | 5.30 | 58.00 | 89.00 | 38.00 | 7.80 | 1.40 | 11.90 | 78.00 |
| ERR2240444 | TVZ | Tikitere | 22 | -38.063 | 176.3609 | 60.6 | 5.53 | 283 | 5.30 | 131.00 | 66.00 | 38.00 | 8.10 | 1.50 | 9.30 | 82.00 |
| ERR2240462 | TVZ | Tikitere | 23 | -38.0609 | 176.3594 | 66.8 | 2.00 | 3891 | 4.00 | 875.00 | 30.00 | 43.00 | 8.90 | 2.80 | 10.60 | 105.00 |
| ERR2240495 | TVZ | Tikitere | 24 | -38.0642 | 176.361 | 44.4 | 2.00 | 5297 | 3.90 | 1309.00 | 66.00 | 32.00 | 10.90 | 2.40 | 13.30 | 90.00 |
| ERR2240498 | TVZ | Tikitere | 25 | -38.0645 | 176.361 | 45.3 | 2.30 | 2816 | 4.30 | 660.00 | 34.00 | 27.00 | 7.00 | 3.10 | 21.00 | 69.00 |
| ERR2240527 | TVZ | Tokaanu | 1 | -38.9675 | 175.7645 | 46.0 | 7.14 | 10120 | 3234.00 | 66.00 | 33.00 | 1821.00 | 156.00 | 0.24 | 42.00 | 122.00 |
| ERR2240528 | TVZ | Tokaanu | 2 | -38.9676 | 175.7644 | 55.7 | 6.82 | 10420 | 3180.00 | 66.00 | 42.00 | 1827.00 | 156.00 | 0.29 | 41.00 | 121.00 |
| ERR2240529 | TVZ | Tokaanu | 3 | -38.9679 | 175.7646 | 87.1 | 6.75 | 10340 | 3152.00 | 67.00 | 95.00 | 1825.00 | 156.00 | 0.92 | 47.00 | 160.00 |
| ERR2240530 | TVZ | Tokaanu | 4 | -38.9679 | 175.7647 | 72.7 | 7.35 | 10650 | 3213.00 | 68.00 | 78.00 | 1817.00 | 159.00 | 0.80 | 45.00 | 160.00 |
| ERR2240531 | TVZ | Tokaanu | 5 | -38.968 | 175.7644 | 82.9 | 7.31 | 10350 | 3158.00 | 65.00 | 59.00 | 1875.00 | 165.00 | 0.14 | 44.00 | 161.00 |
| ERR2240532 | TVZ | Tokaanu | 6 | -38.9679 | 175.7644 | 41.0 | 6.47 | 9823 | 2954.00 | 65.00 | 96.00 | 1675.00 | 146.00 | 0.63 | 40.00 | 147.00 |
| ERR2240533 | TVZ | Tokaanu | 7 | -38.968 | 175.7644 | 66.7 | 6.79 | 10440 | 3228.00 | 69.00 | 90.00 | 1893.00 | 163.00 | 0.83 | 47.00 | 174.00 |
| ERR2240578 | TVZ | Tokaanu | 8 | -38.9683 | 175.7634 | 41.3 | 5.58 | 5443 | 1709.00 | 58.00 | 41.00 | 967.00 | 82.00 | 1.50 | 28.00 | 74.00 |
| ERR2240579 | TVZ | Tokaanu | 9 | -38.9684 | 175.7633 | 47.2 | 5.68 | 8115 | 2519.00 | 72.00 | 23.00 | 1411.00 | 125.00 | 0.50 | 34.00 | 125.00 |
| ERR2240580 | TVZ | Tokaanu | 10 | -38.9684 | 175.7631 | 48.0 | 6.79 | 9744 | 2953.00 | 63.00 | 52.00 | 1697.00 | 135.00 | 0.27 | 39.00 | 117.00 |
| ERR2240581 | TVZ | Tokaanu | 11 | -38.9684 | 175.763 | 53.1 | 6.09 | 10020 | 3042.00 | 74.00 | 60.00 | 1715.00 | 139.00 | 0.34 | 45.00 | 139.00 |
| ERR2240584 | TVZ | Tokaanu | 12 | -38.9686 | 175.7627 | 51.5 | 5.92 | 5753 | 2036.00 | 51.00 | 47.00 | 1132.00 | 83.00 | 0.97 | 35.00 | 97.00 |
| ERR2240619 | TVZ | Tokaanu | 13 | -38.9683 | 175.7622 | 51.9 | 6.23 | 5291 | 1563.00 | 28.00 | 48.00 | 841.00 | 68.00 | 1.10 | 22.00 | 72.00 |
| ERR2240622 | TVZ | Tokaanu | 14 | -38.9682 | 175.7624 | 49.4 | 6.71 | 6735 | 1989.00 | 49.00 | 56.00 | 1151.00 | 86.00 | 1.90 | 43.00 | 101.00 |
| ERR2240623 | TVZ | Tokaanu | 15 | -38.9688 | 175.7624 | 59.9 | 6.61 | 4010 | 1100.00 | 29.00 | 169.00 | 625.00 | 52.00 | 4.20 | 50.00 | 119.00 |
| ERR2240625 | TVZ | Tokaanu | 16 | -38.9682 | 175.7625 | 45.4 | 6.18 | 9044 | 2677.00 | 58.00 | 38.00 | 1551.00 | 121.00 | 1.70 | 39.00 | 116.00 |
| ERR2240903 | TVZ | Waikite | 1 | -38.3279 | 176.2978 | 48.8 | 8.47 | 873 | 125.00 | 27.00 | 244.00 | 199.00 | 11.80 | 0.92 | 11.00 | 92.00 |
| ERR2240904 | TVZ | Waikite | 2 | -38.3281 | 176.3014 | 65.1 | 8.29 | 1188 | 140.00 | 35.00 | 312.00 | 285.00 | 10.80 | 0.60 | 13.00 | 94.00 |
| ERR2240905 | TVZ | Waikite | 3 | -38.3269 | 176.3049 | 96.5 | 7.27 | 1247 | 140.00 | 34.00 | 354.00 | 283.00 | 11.40 | 0.52 | 14.60 | 97.00 |
| ERR2240906 | TVZ | Waikite | 4 | -38.3269 | 176.3049 | 93.3 | 7.49 | 1298 | 142.00 | 35.00 | 352.00 | 276.00 | 11.50 | 0.89 | 17.00 | 96.00 |
| ERR2240907 | TVZ | Waikite | 5 | -38.3269 | 176.3045 | 88.8 | 7.59 | 1231 | 137.00 | 35.00 | 329.00 | 285.00 | 11.20 | 0.66 | 16.00 | 97.00 |
| ERR2240908 | TVZ | Waikite | 6 | -38.327 | 176.3037 | 93.2 | 8.14 | 1233 | 141.00 | 35.00 | 311.00 | 160.00 | 6.00 | 0.41 | 7.90 | 54.00 |
| ERR2240909 | TVZ | Waikite | 7 | -38.327 | 176.3035 | 55.6 | 8.75 | 1318 | 151.00 | 37.00 | 302.00 | 288.00 | 10.90 | 0.47 | 9.00 | 98.00 |
| ERR2240910 | TVZ | Waikite | 8 | -38.3272 | 176.3031 | 48.2 | 8.90 | 1330 | 153.00 | 38.00 | 298.00 | 292.00 | 11.00 | 0.44 | 9.00 | 99.00 |
| ERR2240164 | TVZ | Waimangu | 1 | -38.2829 | 176.3989 | 98.8 | 7.65 | 2155 | 471.00 | 150.00 | 103.00 | 670.00 | 89.00 | 2.10 | 28.00 | 283.00 |
| ERR2240165 | TVZ | Waimangu | 2 | -38.2828 | 176.3989 | 88.6 | 8.22 | 2218 | 483.00 | 152.00 | 88.00 | 576.00 | 79.00 | 1.30 | 23.00 | 250.00 |
| ERR2240166 | TVZ | Waimangu | 3 | -38.2829 | 176.3989 | 95.2 | 6.49 | 1873 | 371.00 | 164.00 | 133.00 | 530.00 | 74.00 | 4.20 | 37.00 | 236.00 |
| ERR2240171 | TVZ | Waimangu | 4 | -38.2827 | 176.3986 | 97.2 | 8.78 | 2699 | 595.00 | 123.00 | 75.00 | 790.00 | 113.00 | 0.21 | 19.40 | 368.00 |
| ERR2240215 | TVZ | Waimangu | 5 | -38.2828 | 176.3977 | 70.0 | 4.72 | 2678 | 678.00 | 178.00 | 31.00 | 746.00 | 85.00 | 6.10 | 29.00 | 245.00 |
| ERR2240218 | TVZ | Waimangu | 6 | -38.2828 | 176.3976 | 89.5 | 6.76 | 2888 | 775.00 | 105.00 | 45.00 | 690.00 | 78.00 | 1.90 | 21.00 | 240.00 |
| ERR2240221 | TVZ | Waimangu | 7 | -38.2829 | 176.3974 | 86.9 | 5.81 | 2422 | 551.00 | 219.00 | 51.00 | 677.00 | 60.00 | 2.90 | 16.40 | 217.00 |
| ERR2240234 | TVZ | Waimangu | 8 | -38.2826 | 176.3989 | 62.0 | 6.35 | 2173 | 563.00 | 70.00 | 58.00 | 648.00 | 106.00 | 12.70 | 35.00 | 269.00 |
| ERR2240235 | TVZ | Waimangu | 9 | -38.2826 | 176.3989 | 51.9 | 6.53 | 2022 | 462.00 | 171.00 | 50.00 | 526.00 | 97.00 | 17.80 | 61.00 | 233.00 |
| ERR2240236 | TVZ | Waimangu | 10 | -38.2825 | 176.399 | 59.7 | 6.04 | 2042 | 510.00 | 68.00 | 91.00 | 550.00 | 87.00 | 13.90 | 42.00 | 215.00 |
| ERR2240237 | TVZ | Waimangu | 11 | -38.283 | 176.3994 | 89.1 | 8.02 | 2402 | 528.00 | 135.00 | 174.00 | 729.00 | 100.00 | 0.20 | 17.20 | 301.00 |
| ERR2240300 | TVZ | Waimangu | 12 | -38.283 | 176.3997 | 70.2 | 6.75 | 1445 | 278.00 | 90.00 | 148.00 | 217.00 | 31.00 | 1.00 | 7.20 | 121.00 |
| ERR2240307 | TVZ | Waimangu | 13 | -38.2833 | 176.3999 | 62.1 | 8.66 | 1923 | 465.00 | 133.00 | 96.00 | 326.00 | 43.00 | 0.36 | 7.10 | 104.00 |
| ERR2240314 | TVZ | Waimangu | 14 | -38.2831 | 176.3968 | 90.5 | 5.88 | 2256 | 550.00 | 205.00 | 52.00 | 388.00 | 33.00 | 1.50 | 8.20 | 130.00 |
| ERR2240337 | TVZ | Waimangu | 15 | -38.2784 | 176.4085 | 93.6 | 7.60 | 2355 | 583.00 | 94.00 | 160.00 | 416.00 | 45.00 | 0.19 | 5.60 | 191.00 |
| ERR2240342 | TVZ | Waimangu | 16 | -38.2785 | 176.4087 | 70.7 | 7.23 | 2546 | 615.00 | 99.00 | 172.00 | 413.00 | 43.00 | 0.33 | 7.00 | 167.00 |
| ERR2240356 | TVZ | Waimangu | 17 | -38.2793 | 176.4094 | 60.6 | 8.98 | 2595 | 704.00 | 79.00 | 115.00 | 474.00 | 41.00 | 0.19 | 8.70 | 136.00 |
| ERR2240360 | TVZ | Waimangu | 18 | -38.2802 | 176.4074 | 88.1 | 8.13 | 1408 | 189.00 | 97.00 | 298.00 | 239.00 | 27.00 | 2.70 | 14.10 | 139.00 |
| ERR2240361 | TVZ | Waimangu | 19 | -38.2801 | 176.4074 | 73.6 | 7.89 | 1673 | 286.00 | 106.00 | 220.00 | 259.00 | 32.00 | 0.28 | 13.80 | 125.00 |
| ERR2240535 | TVZ | Waimangu | 20 | -38.2828 | 176.3987 | 97.6 | 7.85 | 2446 | 536.00 | 151.00 | 89.00 | 355.00 | 48.00 | 3.00 | 17.10 | 144.00 |
| ERR2240537 | TVZ | Waimangu | 21 | -38.2828 | 176.3986 | 91.0 | 7.56 | 2572 | 556.00 | 164.00 | 72.00 | 404.00 | 49.00 | 2.30 | 17.60 | 147.00 |
| ERR2240836 | TVZ | Waimangu | 22 | -38.27 | 176.4175 | 98.0 | 9.33 | 3343 | 691.00 | 133.00 | 137.00 | 620.00 | 35.00 | 0.20 | 5.00 | 204.00 |
| ERR2240837 | TVZ | Waimangu | 23 | -38.2686 | 176.4195 | 90.0 | 6.88 | 2112 | 336.00 | 92.00 | 221.00 | 312.00 | 29.00 | 4.00 | 11.30 | 107.00 |
| ERR2240839 | TVZ | Waimangu | 24 | -38.2641 | 176.4188 | 96.9 | 8.56 | 315 | 42.00 | 23.00 | 32.00 | 48.00 | 3.70 | 0.56 | 6.20 | 40.00 |
| ERR2240176 | TVZ | Waiotapu | 1 | -38.3582 | 176.3695 | 67.2 | 4.80 | 5007 | 1313.00 | 209.00 | 76.00 | 1069.00 | 139.00 | 0.69 | 36.00 | 158.00 |
| ERR2240206 | TVZ | Waiotapu | 2 | -38.3611 | 176.3688 | 48.8 | 2.41 | 3660 | 490.00 | 512.00 | 28.00 | 522.00 | 97.00 | 5.60 | 38.00 | 179.00 |
| ERR2240318 | TVZ | Waiotapu | 3 | -38.3618 | 176.3684 | 74.8 | 5.12 | 2340 | 586.00 | 260.00 | 61.00 | 345.00 | 34.00 | 0.61 | 18.20 | 126.00 |
| ERR2240319 | TVZ | Waiotapu | 4 | -38.3618 | 176.3685 | 66.0 | 5.06 | 2361 | 594.00 | 265.00 | 74.00 | 353.00 | 35.00 | 0.47 | 15.00 | 127.00 |
| ERR2240320 | TVZ | Waiotapu | 5 | -38.3618 | 176.3685 | 89.5 | 5.38 | 2229 | 564.00 | 254.00 | 57.00 | 337.00 | 33.00 | 0.46 | 14.40 | 121.00 |
| ERR2240322 | TVZ | Waiotapu | 6 | -38.3618 | 176.3683 | 41.3 | 2.59 | 3824 | 661.00 | 458.00 | 189.00 | 401.00 | 42.00 | 0.57 | 18.20 | 124.00 |
| ERR2240327 | TVZ | Waiotapu | 7 | -38.3618 | 176.3683 | 43.0 | 5.28 | 2802 | 769.00 | 123.00 | 226.00 | 456.00 | 40.00 | 1.10 | 25.00 | 135.00 |
| ERR2240334 | TVZ | Waiotapu | 8 | -38.362 | 176.368 | 45.8 | 2.04 | 6345 | 330.00 | 1420.00 | 61.00 | 217.00 | 28.00 | 2.60 | 22.00 | 138.00 |
| ERR2240380 | TVZ | Waiotapu | 9 | -38.3622 | 176.3679 | 75.2 | 2.17 | 5681 | 609.00 | 822.00 | 26.00 | 375.00 | 37.00 | 2.50 | 25.00 | 140.00 |
| ERR2240384 | TVZ | Waiotapu | 10 | -38.3619 | 176.3694 | 62.3 | 4.93 | 2486 | 673.00 | 83.00 | 44.00 | 369.00 | 60.00 | 0.36 | 15.80 | 191.00 |
| ERR2240388 | TVZ | Waiotapu | 11 | -38.3629 | 176.3688 | 40.8 | 2.40 | 4196 | 510.00 | 526.00 | 43.00 | 296.00 | 49.00 | 2.80 | 26.00 | 121.00 |
| ERR2240389 | TVZ | Waiotapu | 12 | -38.3613 | 176.3705 | 66.1 | 2.60 | 2730 | 228.00 | 518.00 | 110.00 | 183.00 | 33.00 | 2.70 | 24.00 | 104.00 |
| ERR2240563 | TVZ | Waiotapu | 13 | -38.3506 | 176.377 | 82.3 | 2.31 | 3426 | 357.00 | 512.00 | 24.00 | 230.00 | 41.00 | 1.60 | 14.60 | 140.00 |
| ERR2240924 | TVZ | Waiotapu | 14 | -38.3622 | 176.3699 | 92.8 | 6.50 | 1361 | 1.90 | 367.00 | 26.00 | 23.00 | 11.80 | 2.20 | 40.00 | 70.00 |
| ERR2240925 | TVZ | Waiotapu | 15 | -38.3622 | 176.3701 | 83.4 | 5.55 | 2340 | 0.32 | 613.00 | 44.00 | 1.70 | 1.60 | 0.32 | 4.60 | 36.00 |
| ERR2240129 | TVZ | Wairakei-Tauhara | 1 | -38.6325 | 176.0981 | 44.6 | 6.65 | 1935 | 477.00 | 6.40 | 214.00 | 590.00 | 90.00 | 27.00 | 51.00 | 157.00 |
| ERR2240519 | TVZ | Wairakei-Tauhara | 2 | -38.7002 | 176.0845 | 63.1 | 6.39 | 1069 | 105.00 | 128.00 | 251.00 | 153.00 | 27.00 | 3.90 | 23.00 | 113.00 |
| ERR2240520 | TVZ | Wairakei-Tauhara | 3 | -38.7002 | 176.0844 | 55.3 | 6.80 | 1120 | 106.00 | 126.00 | 241.00 | 229.00 | 30.00 | 5.40 | 25.00 | 114.00 |
| ERR2240521 | TVZ | Wairakei-Tauhara | 4 | -38.7003 | 176.0842 | 45.4 | 6.64 | 1179 | 127.00 | 112.00 | 262.00 | 163.00 | 31.00 | 3.70 | 24.00 | 105.00 |
| ERR2240522 | TVZ | Wairakei-Tauhara | 5 | -38.7003 | 176.0843 | 61.6 | 6.93 | 1274 | 143.00 | 112.00 | 287.00 | 176.00 | 35.00 | 3.90 | 26.00 | 114.00 |
| ERR2240523 | TVZ | Wairakei-Tauhara | 6 | -38.7003 | 176.0843 | 57.0 | 7.00 | 1232 | 140.00 | 112.00 | 274.00 | 172.00 | 35.00 | 4.50 | 28.00 | 117.00 |
| ERR2240524 | TVZ | Wairakei-Tauhara | 7 | -38.7003 | 176.0842 | 51.9 | 7.01 | 1163 | 129.00 | 115.00 | 249.00 | 163.00 | 32.00 | 3.70 | 23.00 | 115.00 |
| ERR2240525 | TVZ | Wairakei-Tauhara | 8 | -38.7003 | 176.0842 | 60.5 | 6.35 | 1141 | 122.00 | 114.00 | 276.00 | 156.00 | 32.00 | 3.80 | 25.00 | 113.00 |
| ERR2240526 | TVZ | Wairakei-Tauhara | 9 | -38.7017 | 176.085 | 67.0 | 6.28 | 1376 | 163.00 | 100.00 | 343.00 | 188.00 | 35.00 | 4.10 | 26.00 | 118.00 |
| ERR2240543 | TVZ | Wairakei-Tauhara | 10 | -38.702 | 176.0852 | 45.0 | 6.71 | 1350 | 167.00 | 100.00 | 327.00 | 199.00 | 37.00 | 4.00 | 27.00 | 123.00 |
| ERR2240544 | TVZ | Wairakei-Tauhara | 11 | -38.7032 | 176.0865 | 51.2 | 6.38 | 1682 | 224.00 | 72.00 | 470.00 | 255.00 | 36.00 | 4.70 | 28.00 | 120.00 |
| ERR2240545 | TVZ | Wairakei-Tauhara | 12 | -38.7044 | 176.087 | 56.7 | 6.49 | 1997 | 343.00 | 73.00 | 355.00 | 306.00 | 32.00 | 4.50 | 24.00 | 128.00 |
| ERR2240546 | TVZ | Wairakei-Tauhara | 13 | -38.7045 | 176.087 | 59.8 | 6.37 | 1927 | 325.00 | 71.00 | 354.00 | 286.00 | 31.00 | 4.70 | 25.00 | 113.00 |
| ERR2240547 | TVZ | Wairakei-Tauhara | 14 | -38.7045 | 176.087 | 54.7 | 6.51 | 2022 | 368.00 | 74.00 | 353.00 | 310.00 | 33.00 | 4.90 | 23.00 | 126.00 |
| ERR2240548 | TVZ | Wairakei-Tauhara | 15 | -38.7056 | 176.0865 | 55.0 | 7.15 | 1777 | 298.00 | 81.00 | 280.00 | 267.00 | 27.00 | 4.80 | 22.00 | 115.00 |
| ERR2240549 | TVZ | Wairakei-Tauhara | 16 | -38.7058 | 176.0867 | 59.9 | 6.41 | 1753 | 284.00 | 82.00 | 321.00 | 271.00 | 26.00 | 5.30 | 23.00 | 113.00 |
| ERR2240550 | TVZ | Wairakei-Tauhara | 17 | -38.708 | 176.0875 | 56.2 | 6.93 | 1857 | 318.00 | 53.00 | 329.00 | 278.00 | 29.00 | 4.80 | 22.00 | 119.00 |
| ERR2240553 | TVZ | Wairakei-Tauhara | 18 | -38.6706 | 176.0894 | 41.7 | 6.70 | 770 | 14.20 | 235.00 | 96.00 | 109.00 | 19.80 | 6.00 | 20.00 | 109.00 |
| ERR2240555 | TVZ | Wairakei-Tauhara | 19 | -38.6741 | 176.0998 | 71.6 | 6.90 | 1164 | 8.30 | 427.00 | 62.00 | 155.00 | 34.00 | 7.80 | 30.00 | 168.00 |
| ERR2240990 | TVZ | Wairakei-Tauhara | 20 | -38.7065 | 176.0874 | 72.5 | 6.28 | 1899 | 344.00 | 56.00 | 336.00 | 307.00 | 34.00 | 3.60 | 22.00 | 126.00 |
| ERR2240991 | TVZ | Wairakei-Tauhara | 21 | -38.7064 | 176.0876 | 40.3 | 8.21 | 1867 | 336.00 | 70.00 | 267.00 | 314.00 | 35.00 | 5.00 | 29.00 | 118.00 |
| ERR2240513 | TVZ | Whakaari (White Island) | 1 | -37.5253 | 177.1904 | 84.0 | 5.41 | 1471 | 73.00 | 804.00 | 57.00 | 42.00 | 12.40 | 51.00 | 63.00 | 101.00 |
| ERR2240565 | TVZ | Whakaari (White Island) | 2 | -37.5257 | 177.1903 | 68.0 | 3.13 | 4151 | 128.00 | 1969.00 | 60.00 | 240.00 | 29.00 | 171.00 | 381.00 | 125.00 |
| ERR2240567 | TVZ | Whakaari (White Island) | 3 | -37.5256 | 177.1902 | 41.4 | 3.05 | 4299 | 126.00 | 2131.00 | 104.00 | 140.00 | 25.00 | 173.00 | 381.00 | 133.00 |

Supplementary Table 2 Observed richness and Shannon indexes of the analyzed hot springs.

| **SRA number** | **Area** | **Geothermal field** | **N°** | **Observed** | **Shannon** |
| --- | --- | --- | --- | --- | --- |
| SRR18711309 | APVF | El Tatio | 1 | 66 | 2.91 |
| SRR18711297 | APVF | El Tatio | 2 | 128 | 3.37 |
| SRR18711284 | APVF | El Tatio | 3 | 78 | 2.97 |
| SRR18711281 | APVF | El Tatio | 4 | 85 | 2.66 |
| SRR18711307 | APVF | El Tatio | 5 | 91 | 3.25 |
| SRR18711304 | APVF | El Tatio | 6 | 129 | 3.35 |
| SRR18711301 | APVF | El Tatio | 7 | 84 | 3.03 |
| SRR18711299 | APVF | El Tatio | 8 | 94 | 3.58 |
| SRR18711295 | APVF | El Tatio | 9 | 65 | 2.81 |
| SRR18711292 | APVF | El Tatio | 10 | 245 | 3.33 |
| SRR18711289 | APVF | El Tatio | 11 | 166 | 3.50 |
| SRR8420032 | YPVF | Yellowstone | 1 | 129 | 3.50 |
| SRR8420033 | YPVF | Yellowstone | 8 | 222 | 3.55 |
| SRR8420034 | YPVF | Yellowstone | 9 | 197 | 4.11 |
| SRR8420035 | YPVF | Yellowstone | 10 | 63 | 2.90 |
| SRR8420036 | YPVF | Yellowstone | 11 | 578 | 4.71 |
| SRR8420037 | YPVF | Yellowstone | 12 | 199 | 3.39 |
| SRR8420038 | YPVF | Yellowstone | 13 | 391 | 4.39 |
| SRR8420039 | YPVF | Yellowstone | 14 | 118 | 3.88 |
| SRR8420040 | YPVF | Yellowstone | 15 | 89 | 3.39 |
| SRR8420041 | YPVF | Yellowstone | 16 | 156 | 3.87 |
| SRR8420042 | YPVF | Yellowstone | 2 | 291 | 4.20 |
| SRR8420043 | YPVF | Yellowstone | 3 | 170 | 4.20 |
| SRR8420044 | YPVF | Yellowstone | 4 | 104 | 3.18 |
| SRR8420045 | YPVF | Yellowstone | 17 | 79 | 3.26 |
| SRR8420046 | YPVF | Yellowstone | 5 | 117 | 3.76 |
| SRR8420047 | YPVF | Yellowstone | 6 | 63 | 1.98 |
| SRR8420048 | YPVF | Yellowstone | 7 | 192 | 3.75 |
| SRR8420049 | YPVF | Yellowstone | 18 | 266 | 4.38 |
| SRR8420050 | YPVF | Yellowstone | 19 | 71 | 3.10 |
| SRR8420052 | YPVF | Yellowstone | 20 | 38 | 1.89 |
| SRR8420053 | YPVF | Yellowstone | 21 | 74 | 2.28 |
| SRR8420054 | YPVF | Yellowstone | 22 | 111 | 3.54 |
| SRR8420055 | YPVF | Yellowstone | 23 | 122 | 3.79 |
| SRR8420056 | YPVF | Yellowstone | 24 | 63 | 2.93 |
| SRR8420057 | YPVF | Yellowstone | 25 | 87 | 3.23 |
| SRR10580885 | ETPGB | Batang | 1 | 115 | 2.37 |
| SRR10580888 | ETPGB | Batang | 2 | 110 | 2.74 |
| SRR10580889 | ETPGB | Batang | 3 | 105 | 2.70 |
| SRR10580891 | ETPGB | Batang | 4 | 159 | 3.37 |
| SRR10580894 | ETPGB | Batang | 5 | 204 | 3.06 |
| SRR10580896 | ETPGB | Batang | 6 | 638 | 5.14 |
| SRR10580898 | ETPGB | Batang | 7 | 196 | 1.63 |
| SRR10580884 | ETPGB | Kangding | 1 | 127 | 1.61 |
| SRR10580886 | ETPGB | Kangding | 2 | 91 | 1.99 |
| SRR10580890 | ETPGB | Kangding | 3 | 479 | 3.95 |
| SRR10580892 | ETPGB | Kangding | 4 | 22 | 0.58 |
| SRR10580895 | ETPGB | Kangding | 5 | 167 | 3.69 |
| SRR10580897 | ETPGB | Kangding | 6 | 55 | 1.00 |
| SRR10580899 | ETPGB | Kangding | 7 | 155 | 2.50 |
| ERR2240344 | TVZ | Orakei Korako | 1 | 180 | 3.49 |
| ERR2240345 | TVZ | Orakei Korako | 2 | 322 | 3.81 |
| ERR2240346 | TVZ | Orakei Korako | 3 | 121 | 2.44 |
| ERR2240347 | TVZ | Orakei Korako | 4 | 27 | 1.50 |
| ERR2240348 | TVZ | Orakei Korako | 5 | 131 | 2.66 |
| ERR2240349 | TVZ | Orakei Korako | 6 | 47 | 1.63 |
| ERR2241038 | TVZ | Orakei Korako | 7 | 249 | 3.39 |
| ERR2241039 | TVZ | Orakei Korako | 8 | 16 | 0.83 |
| ERR2241040 | TVZ | Orakei Korako | 9 | 127 | 3.35 |
| ERR2241041 | TVZ | Orakei Korako | 10 | 205 | 3.52 |
| ERR2241044 | TVZ | Orakei Korako | 11 | 12 | 0.82 |
| ERR2241045 | TVZ | Orakei Korako | 12 | 24 | 0.98 |
| ERR2241046 | TVZ | Orakei Korako | 13 | 112 | 2.46 |
| ERR2241047 | TVZ | Orakei Korako | 14 | 61 | 1.70 |
| ERR2241048 | TVZ | Orakei Korako | 15 | 122 | 1.90 |
| ERR2240422 | TVZ | Rotorua | 1 | 65 | 2.82 |
| ERR2240473 | TVZ | Rotorua | 2 | 94 | 2.23 |
| ERR2240483 | TVZ | Rotorua | 3 | 15 | 1.70 |
| ERR2240508 | TVZ | Rotorua | 4 | 85 | 2.83 |
| ERR2240516 | TVZ | Rotorua | 5 | 71 | 2.88 |
| ERR2240588 | TVZ | Rotorua | 6 | 132 | 2.85 |
| ERR2240589 | TVZ | Rotorua | 7 | 38 | 2.46 |
| ERR2240629 | TVZ | Rotorua | 8 | 122 | 2.45 |
| ERR2240637 | TVZ | Rotorua | 9 | 44 | 1.40 |
| ERR2240654 | TVZ | Rotorua | 10 | 52 | 1.99 |
| ERR2240662 | TVZ | Rotorua | 11 | 43 | 1.97 |
| ERR2240672 | TVZ | Rotorua | 12 | 145 | 3.32 |
| ERR2240680 | TVZ | Rotorua | 13 | 24 | 1.32 |
| ERR2240707 | TVZ | Rotorua | 14 | 67 | 2.83 |
| ERR2240712 | TVZ | Rotorua | 15 | 23 | 2.05 |
| ERR2240716 | TVZ | Rotorua | 16 | 208 | 3.49 |
| ERR2240728 | TVZ | Rotorua | 17 | 233 | 4.11 |
| ERR2240756 | TVZ | Rotorua | 18 | 15 | 1.58 |
| ERR2240786 | TVZ | Rotorua | 19 | 37 | 1.80 |
| ERR2240797 | TVZ | Rotorua | 20 | 18 | 1.36 |
| ERR2240800 | TVZ | Rotorua | 21 | 28 | 1.23 |
| ERR2240809 | TVZ | Rotorua | 22 | 58 | 2.37 |
| ERR2240825 | TVZ | Rotorua | 23 | 93 | 2.90 |
| ERR2240865 | TVZ | Rotorua | 24 | 29 | 2.44 |
| ERR2240874 | TVZ | Rotorua | 25 | 85 | 3.34 |
| ERR2240258 | TVZ | Tikitere | 1 | 41 | 1.71 |
| ERR2240260 | TVZ | Tikitere | 2 | 28 | 1.66 |
| ERR2240270 | TVZ | Tikitere | 3 | 50 | 2.95 |
| ERR2240271 | TVZ | Tikitere | 4 | 45 | 3.13 |
| ERR2240272 | TVZ | Tikitere | 5 | 114 | 3.34 |
| ERR2240273 | TVZ | Tikitere | 6 | 22 | 1.17 |
| ERR2240276 | TVZ | Tikitere | 7 | 47 | 2.94 |
| ERR2240284 | TVZ | Tikitere | 8 | 18 | 2.20 |
| ERR2240285 | TVZ | Tikitere | 9 | 19 | 2.27 |
| ERR2240286 | TVZ | Tikitere | 10 | 20 | 1.64 |
| ERR2240290 | TVZ | Tikitere | 11 | 43 | 2.21 |
| ERR2240292 | TVZ | Tikitere | 12 | 60 | 2.93 |
| ERR2240393 | TVZ | Tikitere | 13 | 37 | 2.35 |
| ERR2240400 | TVZ | Tikitere | 14 | 42 | 3.02 |
| ERR2240411 | TVZ | Tikitere | 15 | 53 | 1.81 |
| ERR2240414 | TVZ | Tikitere | 16 | 30 | 2.30 |
| ERR2240417 | TVZ | Tikitere | 17 | 12 | 1.75 |
| ERR2240434 | TVZ | Tikitere | 18 | 44 | 1.84 |
| ERR2240436 | TVZ | Tikitere | 19 | 44 | 2.83 |
| ERR2240439 | TVZ | Tikitere | 20 | 36 | 2.17 |
| ERR2240442 | TVZ | Tikitere | 21 | 49 | 2.01 |
| ERR2240444 | TVZ | Tikitere | 22 | 26 | 1.03 |
| ERR2240462 | TVZ | Tikitere | 23 | 66 | 2.59 |
| ERR2240495 | TVZ | Tikitere | 24 | 49 | 3.54 |
| ERR2240498 | TVZ | Tikitere | 25 | 17 | 1.94 |
| ERR2240527 | TVZ | Tokaanu | 1 | 80 | 2.27 |
| ERR2240528 | TVZ | Tokaanu | 2 | 48 | 2.65 |
| ERR2240529 | TVZ | Tokaanu | 3 | 18 | 0.14 |
| ERR2240530 | TVZ | Tokaanu | 4 | 75 | 1.01 |
| ERR2240531 | TVZ | Tokaanu | 5 | 102 | 3.30 |
| ERR2240532 | TVZ | Tokaanu | 6 | 107 | 2.44 |
| ERR2240533 | TVZ | Tokaanu | 7 | 61 | 1.62 |
| ERR2240578 | TVZ | Tokaanu | 8 | 361 | 3.72 |
| ERR2240579 | TVZ | Tokaanu | 9 | 239 | 3.26 |
| ERR2240580 | TVZ | Tokaanu | 10 | 159 | 2.71 |
| ERR2240581 | TVZ | Tokaanu | 11 | 209 | 3.67 |
| ERR2240584 | TVZ | Tokaanu | 12 | 241 | 3.51 |
| ERR2240619 | TVZ | Tokaanu | 13 | 264 | 4.77 |
| ERR2240622 | TVZ | Tokaanu | 14 | 353 | 4.62 |
| ERR2240623 | TVZ | Tokaanu | 15 | 72 | 3.15 |
| ERR2240625 | TVZ | Tokaanu | 16 | 408 | 4.44 |
| ERR2240903 | TVZ | Waikite | 1 | 271 | 3.68 |
| ERR2240904 | TVZ | Waikite | 2 | 125 | 3.71 |
| ERR2240905 | TVZ | Waikite | 3 | 198 | 3.85 |
| ERR2240906 | TVZ | Waikite | 4 | 108 | 1.98 |
| ERR2240907 | TVZ | Waikite | 5 | 529 | 4.61 |
| ERR2240908 | TVZ | Waikite | 6 | 257 | 4.89 |
| ERR2240909 | TVZ | Waikite | 7 | 110 | 3.59 |
| ERR2240910 | TVZ | Waikite | 8 | 145 | 4.07 |
| ERR2240164 | TVZ | Waimangu | 1 | 101 | 2.97 |
| ERR2240165 | TVZ | Waimangu | 2 | 130 | 2.10 |
| ERR2240166 | TVZ | Waimangu | 3 | 500 | 2.35 |
| ERR2240171 | TVZ | Waimangu | 4 | 904 | 3.39 |
| ERR2240215 | TVZ | Waimangu | 5 | 115 | 2.79 |
| ERR2240218 | TVZ | Waimangu | 6 | 218 | 2.45 |
| ERR2240221 | TVZ | Waimangu | 7 | 117 | 2.76 |
| ERR2240234 | TVZ | Waimangu | 8 | 197 | 3.32 |
| ERR2240235 | TVZ | Waimangu | 9 | 446 | 4.62 |
| ERR2240236 | TVZ | Waimangu | 10 | 256 | 3.28 |
| ERR2240237 | TVZ | Waimangu | 11 | 143 | 2.18 |
| ERR2240300 | TVZ | Waimangu | 12 | 252 | 4.57 |
| ERR2240307 | TVZ | Waimangu | 13 | 126 | 3.74 |
| ERR2240314 | TVZ | Waimangu | 14 | 40 | 2.98 |
| ERR2240337 | TVZ | Waimangu | 15 | 61 | 1.67 |
| ERR2240342 | TVZ | Waimangu | 16 | 147 | 3.89 |
| ERR2240356 | TVZ | Waimangu | 17 | 138 | 2.77 |
| ERR2240360 | TVZ | Waimangu | 18 | 221 | 2.47 |
| ERR2240361 | TVZ | Waimangu | 19 | 221 | 4.01 |
| ERR2240535 | TVZ | Waimangu | 20 | 774 | 4.07 |
| ERR2240537 | TVZ | Waimangu | 21 | 146 | 2.47 |
| ERR2240836 | TVZ | Waimangu | 22 | 121 | 2.53 |
| ERR2240837 | TVZ | Waimangu | 23 | 198 | 4.28 |
| ERR2240839 | TVZ | Waimangu | 24 | 269 | 4.36 |
| ERR2240176 | TVZ | Waiotapu | 1 | 39 | 0.89 |
| ERR2240206 | TVZ | Waiotapu | 2 | 24 | 2.26 |
| ERR2240318 | TVZ | Waiotapu | 3 | 39 | 2.92 |
| ERR2240319 | TVZ | Waiotapu | 4 | 9 | 1.30 |
| ERR2240320 | TVZ | Waiotapu | 5 | 23 | 2.88 |
| ERR2240322 | TVZ | Waiotapu | 6 | 15 | 1.76 |
| ERR2240327 | TVZ | Waiotapu | 7 | 18 | 1.27 |
| ERR2240334 | TVZ | Waiotapu | 8 | 21 | 1.14 |
| ERR2240380 | TVZ | Waiotapu | 9 | 130 | 3.98 |
| ERR2240384 | TVZ | Waiotapu | 10 | 11 | 0.82 |
| ERR2240388 | TVZ | Waiotapu | 11 | 23 | 1.40 |
| ERR2240389 | TVZ | Waiotapu | 12 | 33 | 1.56 |
| ERR2240563 | TVZ | Waiotapu | 13 | 169 | 4.61 |
| ERR2240924 | TVZ | Waiotapu | 14 | 21 | 1.66 |
| ERR2240925 | TVZ | Waiotapu | 15 | 319 | 3.33 |
| ERR2240129 | TVZ | Wairakei-Tauhara | 1 | 191 | 3.09 |
| ERR2240519 | TVZ | Wairakei-Tauhara | 2 | 108 | 3.72 |
| ERR2240520 | TVZ | Wairakei-Tauhara | 3 | 188 | 3.68 |
| ERR2240521 | TVZ | Wairakei-Tauhara | 4 | 194 | 3.16 |
| ERR2240522 | TVZ | Wairakei-Tauhara | 5 | 58 | 2.25 |
| ERR2240523 | TVZ | Wairakei-Tauhara | 6 | 200 | 4.31 |
| ERR2240524 | TVZ | Wairakei-Tauhara | 7 | 202 | 4.45 |
| ERR2240525 | TVZ | Wairakei-Tauhara | 8 | 104 | 3.62 |
| ERR2240526 | TVZ | Wairakei-Tauhara | 9 | 60 | 1.72 |
| ERR2240543 | TVZ | Wairakei-Tauhara | 10 | 101 | 3.27 |
| ERR2240544 | TVZ | Wairakei-Tauhara | 11 | 107 | 2.68 |
| ERR2240545 | TVZ | Wairakei-Tauhara | 12 | 159 | 3.45 |
| ERR2240546 | TVZ | Wairakei-Tauhara | 13 | 244 | 4.63 |
| ERR2240547 | TVZ | Wairakei-Tauhara | 14 | 257 | 4.52 |
| ERR2240548 | TVZ | Wairakei-Tauhara | 15 | 273 | 4.34 |
| ERR2240549 | TVZ | Wairakei-Tauhara | 16 | 208 | 3.88 |
| ERR2240550 | TVZ | Wairakei-Tauhara | 17 | 366 | 4.60 |
| ERR2240553 | TVZ | Wairakei-Tauhara | 18 | 438 | 5.12 |
| ERR2240555 | TVZ | Wairakei-Tauhara | 19 | 225 | 4.24 |
| ERR2240990 | TVZ | Wairakei-Tauhara | 20 | 500 | 5.31 |
| ERR2240991 | TVZ | Wairakei-Tauhara | 21 | 718 | 5.61 |
| ERR2240513 | TVZ | Whakaari (White Island) | 1 | 28 | 1.35 |
| ERR2240565 | TVZ | Whakaari (White Island) | 2 | 33 | 1.27 |
| ERR2240567 | TVZ | Whakaari (White Island) | 3 | 82 | 2.55 |

Supplementary Table 3 PERMANOVA results at temperatures T < 75°C and T > 75°C using as explanatory variables the principal components of the PCA constructed with hydrochemical variables. Distances were based on weighted Unifrac metric. Pr(>F): p-value associated with the F statistic.

| T < 75°C |  | **Principal Component Analysis (PCA)** | | | | | | | | | | **PERMANOVA** | | |
| --- | --- | --- | --- | --- | --- | --- | --- | --- | --- | --- | --- | --- | --- | --- |
|  |  | **T** | **pH** | **Cl^-^** | **SO_4_^2-^** | **HCO_3_^-^** | **Na^+^** | **K^+^** | **Mg^2+^** | **Ca^2+^** | **Si** | **R^2^** | **F-model** | **Pr(>F)** |
|  | PC1 | -0.144 | -0.194 | -0.447 | 0.246 | 0.065 | -0.498 | -0.501 | 0.068 | -0.223 | -0.352 | 0.076 | 5.23 | P < 0.01 |
|  | PC2 | -0.326 | -0.514 | 0.170 | 0.448 | -0.367 | 0.060 | 0.079 | 0.286 | 0.416 | 0.040 | 0.084 | 5.83 | P < 0.01 |
|  | PC3 | -0.079 | 0.209 | -0.045 | -0.229 | 0.467 | -0.010 | -0.039 | 0.673 | 0.437 | -0.179 | 0.016 | 1.07 | P > 0.1 |
|  | PC4 | -0.670 | -0.082 | 0.308 | -0.095 | 0.323 | 0.225 | 0.054 | -0.278 | -0.178 | -0.416 | 0.034 | 2.34 | P < 0.1 |
|  | PC5 | 0.375 | 0.181 | 0.346 | 0.000 | -0.380 | 0.075 | -0.174 | -0.015 | 0.141 | -0.712 | 0.04 | 0.30 | P > 0.1 |
|  | PC6 | -0.460 | 0.254 | -0.100 | -0.526 | -0.610 | -0.141 | -0.095 | 0.158 | -0.055 | 0.094 | 0.018 | 1.25 | P > 0.1 |
|  | PC7 | -0.177 | 0.618 | 0.024 | 0.602 | -0.074 | 0.106 | 0.143 | 0.276 | -0.333 | 0.020 | 0.014 | 0.99 | P > 0.1 |
|  | PC8 | -0.182 | 0.396 | -0.225 | 0.188 | 0.023 | -0.130 | -0.053 | -0.531 | 0.648 | 0.023 | 0.011 | 0.76 | P > 0.1 |
|  | PC9 | 0.032 | -0.082 | -0.385 | -0.054 | -0.081 | -0.211 | 0.808 | -0.001 | -0.020 | -0.371 | 0.006 | 0.41 | P > 0.1 |
|  | PC10 | -0.010 | 0.069 | 0.588 | 0.013 | 0.100 | -0.774 | 0.153 | -0.014 | -0.007 | 0.125 | 0.007 | 0.50 | P > 0.1 |
|  | **Residual** | | | | | | | | | | | **0.728** |  |  |
|  | **Total** | | | | | | | | | | | **1.000** |  |  |
|  |  | **Principal Component Analysis (PCA)** | | | | | | | | | | **PERMANOVA** | | |
|  |  | **T** | **pH** | **Cl^-^** | **SO_4_^2-^** | **HCO_3_^-^** | **Na^+^** | **K^+^** | **Mg^2+^** | **Ca^2+^** | **Si** | **R^2^** | **F-model** | **Pr(>F)** |
| T > 75°C | PC1 | 0.023 | -0.196 | -0.519 | 0.166 | 0.093 | -0.521 | -0.497 | -0.023 | -0.363 | -0.070 | 0.047 | 8.72 | P < 0.001 |
|  | PC2 | -0.029 | -0.462 | 0.007 | 0.593 | -0.250 | -0.007 | 0.014 | 0.456 | 0.379 | 0.142 | 0.075 | 13.93 | P < 0.001 |
|  | PC3 | -0.242 | 0.229 | -0.070 | -0.030 | 0.586 | -0.030 | -0.074 | 0.473 | 0.302 | -0.468 | 0.027 | 4.94 | P < 0.001 |
|  | PC4 | 0.803 | 0.308 | -0.085 | -0.013 | 0.186 | -0.052 | -0.076 | 0.258 | 0.142 | 0.351 | 0.057 | 10.54 | P < 0.001 |
|  | PC5 | -0.526 | 0.255 | -0.151 | -0.195 | 0.012 | -0.140 | -0.007 | 0.248 | 0.016 | 0.718 | 0.023 | 4.38 | P < 0.001 |
|  | PC6 | -0.026 | 0.529 | -0.063 | -0.029 | -0.708 | -0.089 | -0.263 | 0.166 | 0.142 | -0.297 | 0.041 | 7.57 | P < 0.001 |
|  | PC7 | 0.106 | -0.505 | -0.065 | -0.758 | -0.166 | -0.014 | -0.154 | 0.239 | 0.208 | -0.051 | 0.008 | 1.46 | P > 0.1 |
|  | PC8 | 0.081 | 0.010 | -0.250 | -0.071 | -0.137 | -0.366 | 0.786 | 0.262 | -0.247 | -0.162 | 0.051 | 0.95 | P > 0.1 |
|  | PC9 | -0.017 | 0.029 | -0.383 | -0.004 | 0.031 | -0.247 | 0.168 | -0.532 | 0.691 | 0.016 | 0.013 | 2.50 | P < 0.05 |
|  | PC10 | 0.005 | -0.006 | 0.692 | -0.031 | 0.045 | -0.709 | -0.053 | -0.051 | 0.101 | 0.018 | 0.007 | 1.30 | P > 0.1 |
|  | **Residual** | | | | | | | | | | | **0.70** |  |  |
|  | **Total** | | | | | | | | | | | **1.00** |  |  |

Supplementary Table 4 Strategies and analytical techniques used to obtain the 16S rRNA sequences and the chemical analysis of each set of samples.

|  | **AREA** | **Taupo Volcanic Zone** | **Yellowstone** | **Eastern Tibetan Plateau** | **El Tatio at APVC** |
| --- | --- | --- | --- | --- | --- |
|  | **PUBLICATION** | **Power et al. (2018)** | **Hamilton et al. (2019)** | **Guo et al. (2020)** | **This article** |
|  | Nº available SRA files | 925 | 34 | 16 | - |
|  | Nº used samples | 152 | 25 | 14 | 11 |
| **Genomic data** | Source | ENA, NCBI (PRJEB24353) | NCBI (PRJNA513338) | NCBI (SRP234510) | This article |
|  | Region of the 16S rRNA | V4 | V4 | V4 | V4-V5 |
|  | Type of sample | Water | Microbial mat | Water | Microbial mat |
|  | Technology of sequencing | Life Sciences Ion Torrent PGM | MiSeq Illumina 2 x 300-bp | MiSeq Illumina 2 x 250-bp | MiSeq Illumina 2 x 250-bp |
|  | DNA extraction | Modified CTAB method with the PowerMag Microbial DNA Isolation Kit using SwiftMag technology (MoBio Laboratories, Carlsbad, CA, USA). | DNeasy PowerSoil kit (Qiagen, Carlsbad, CA, USA). | FastDNA Spin Kit for Soil (MP Biomedical, Santa Ana, California, USA). | Xanthogenate buffer and phenol:chloroform extraction according to Alcorta et al. (2018). |
|  | Forward primer | F515 (5′-GTGCCAGCMGCCGCGGTAA-3′) | 515F (5'-TCGTCGGCAGCGTCAGATGTGTATAAGAGACAGGTGCCAGCMGCCGCGGTAA-3') | 515F (5′-GTGCCAGCMGCCGCGG-3′) | 515F (5’-GTGYCAGCMGCCGCGGTAA-3') |
|  | Reverse primer | R806 (5′-GGACTACVSGGGTATCTAAT-3′) | 806R (5'-GTCTCGTGGGCTCGGAGATGTGTATAAGAGACAGGGACTACHVGGGTWTCTAAT-3') | 806R (5′-GGACTACHVGGGTWTCTAAT-3′) | 926R (5’-CCGYCAATTYMTTTRAGTTT-3') |
|  | Percentage primer match NR SSU Silva r138.1 (TestPrime) | 9.3 | 71.4 | 72.4 | 83.9 |
| **Hydrochemical data** | Source | 1000springs.org.nz/ | NCBI, Publication | NCBI, Publication | This article |
|  | Anion quantification | Ion Chromatography (Cl^-^, SO_4_^2-^), tritation (HCO_3_^-^), potentiometric tritation (Cl^-^) | Ion Chromatography | Ion Chromatography (Cl^-^, SO_4_^2-^), potentiometric tritation (HCO_3_^-^) | Ion Chromatography (Cl^-^, SO_4_^2-^),  tritation (HCO_3_) |
|  | Cation quantification | Inductively Coupled Plasma Mass Spectroscopy (ICP-MS) | Inductively Coupled Plasma Mass Spectroscopy (ICP-MS) | Inductively Coupled Plasma Optical Emission Spectrometer (ICP-OES) | Flame Atomic Absorption  Spectroscopy |

## Supplementary Text

### Hot springs hydrochemistry (Full-text)

Physicochemical parameters and major ion concentrations of the analyzed hot springs covered a broad hydrochemical spectrum (Supplementary Table 1), which opened the possibility of analyzing microbial communities in different hydrochemical scenarios. Temperatures ranged between 31.5 and 99 °C, pH between 1.5 and 9.9, and electrical conductivity between 236 and 21,000 μS/cm (Figure 2A-C). Ionic concentrations varied as much as 5 orders of magnitude, such as Cl^-^, which ranged from 0.05 mg/l to 7,061 mg/l. SO_4_^2-^ concentrations ranged from 1.6 mg/l to 2,418 mg/l and HCO_3_^-^ values ranged from 0 to 1,228 mg/l. As for cationic concentrations, Na^+^ values reported the broadest range among the samples ranging from 1.7 mg/l and 4,580 mg/l, followed by K^+^ with concentrations between 1.6 mg/l to 508 mg/l. Ca^2+^ and Mg^2+^ values ranged from 0.36 mg/l to 381 mg/l and from 0 to 173 mg/l respectively, and Si concentrations ranged from 14.06 mg/l to 368 mg/l. Zonal dependence was observed for the hydrochemical ranges, with greater homogeneity in El Tatio and ETPGB samples than in YPVF and TVZ, although ETPGB samples covered a wide temperature range, between 37.4°C and 88.2°C.

Samples were plotted on a Piper diagram (Piper, 1944; Figure 2D) according to their major ions concentration. Cl^-^ was the dominant anion in 71% of the samples and Na^+^ was the dominant cation in 92%, meaning that most of the waters were classified as Na-Cl type. Looking at the scale of the study zone, it was noticed that waters in the APVC were all Na-Cl type, while in the ETPGB there were only HCO_3_-type waters. Furthermore, most of the Na-HCO_3_ and Ca-HCO_3_ samples in the dataset belonged to the ETPGB. As for the YPVF and the TVZ samples, they comprised Na-Cl and Na-SO_4_ waters, and a reduced number of Na-HCO_3_.

#### NaCl water

Most of the variance in the water chemistry was explained by Cl^-^, Na^+,^ and K^+^ concentrations (Supplementary Figure 1). From the 202 analyzed hot springs, 107 were classified as NaCl waters (Figure 3). Cl^-^ concentrations at El Tatio were the highest of all samples, reaching 7,062 mg/l, while Tokaanu’s Cl^-^ concentrations reached 3,234 mg/l, being the highest in the TVZ. At El Tatio, water-rock interactions between thermal fluids and Mesozoic to Quaternary volcanic rocks have been proposed (Munoz-Saez et al., 2018), which contribute to the fact that concentrations of Na^+^ and Cl^-^ are more than an order of magnitude higher than those of other ions. High salinities in Tokaanu have been interpreted as a cause of steam loss and surface evaporation processes (60–62). Also, both processes have been described as two of the most important secondary events at El Tatio (Munoz-Saez et al., 2018; Nicolau et al., 2014), which probably also contribute to concentrate the Cl^-^ and many other compounds in the rising water. In contrast, NaCl waters from Wairakei-Tauhara, Waimangu, Rotorua, Orakei-Korako, and Yellowstone had Cl^-^ concentrations below 1,314 mg/l and higher ratios of HCO_3_^-^ to Cl^-^ and SO_4_^2-^ to Cl^-^, which may suggest that other secondary processes are controlling their major element chemistry.

#### Acid-sulfate waters

Of the 60 acid-sulfate water samples (Figure 3), 3 of them were taken at the active White Island volcano. The 3 analyzed samples had pH values between 3.05 and 5.41, and SO_4_^2-^ concentrations between 804 and 1,969 mg/l. Surface fluids of this geothermal system have been shown to contain extremely low pH reaching negative values (63). Fluids sources identified include meteoric water, seawater, and magmatic steam (63–65). The latter has been shown to contribute sulfur more as SO_2_ and less as H_2_S (Christenson et al., 2017) and these inputs are responsible for the low pH values reported (66).

The remaining 57 samples belonged to Yellowstone, Waiotapu, Wairakei-Tauhara, and Tikitere, and their acidity has previously been interpreted as the result of S-rich fluid phase separation and condensation of the steam phase followed, in many cases, by mixing with shallower water (9, 67–70). In addition, Nordstrom et al. (2009) deduced many mixing processes in the YPVF that can explain the SO_4_^2-^ and Cl^-^ concentrations in the analyzed waters. These authors proposed two end members for meteoric water: one consisting of meteoric water only (MO) and another formed by mixing meteoric water with $\mathrm{sulfate}$-rich steam (MG) (Figure 4A). They also proposed three hydrothermal waters: hydrothermal water only (HO), hydrothermal water with subsurface boiling (HB), and hydrothermal water with subsurface boiling and hot gas discharge (HBG). Thus, the highest concentrations of SO_4_^2-^ included in this study (200 mg/l < SO_4_^2-^ < 645 mg/l) could have formed due to mixing of MO and MG (Figure 4A). Intermediate SO_4_^2-^ concentrations (40 mg/l < SO_4_^2-^ < 200 mg/l) may have been HBG or could have been produced due to the mixing of MG with MO. The lowest SO_4_^2-^ concentration (SO_4_^2-^ < 40 mg/l) could have been formed by mixing MO with HO.

On the other hand, hydrochemical trends were observed at Tikitere and Waiotapu, where samples displayed between the SO_4_^2-^ and HCO_3_^-^ vertex of the ternary diagram in Tikitere, and the SO_4_^2-^ and Cl^-^ vertex in Waiotapu (Figure 3). These samples exhibited pH values of 1.57 to 6.48 and 2.31 to 5.55, respectively. Lower pH values were observed to be correlated with higher Eh values (Supplementary Figure 2), leading to relate acidity to shallower fluid circulation. High concentrations of sulfate in these acidic samples also contribute to consider oxidation of reduced S as an important mechanism of acidity. More information on water chemistry would be needed to evaluate the role of water-rock interactions in the formation of these acid-sulfate waters.

At El Tatio, steam-heated waters have been reported (Cortecci et al., 2005; Giggenbach, 1978; Letelier et al., 2021; Munoz-Saez et al., 2018; Tassi et al., 2010) as well as at Tokaanu (Robinson and Sheppard, 1986; Soto et al., 2019), but these samples were not included in the present study.

#### HCO_3_^-^ or CO_2_-rich waters

For Batang, Kangding, Waikite, Waimangu, Tikitere, Wairakei-Tauhara, and Yellowstone, 35 samples were classified as HCO_3_^-^ or CO_2_-rich water. The highest HCO_3_^-^ concentrations were found in the ETPGB (412 mg/l > HCO_3_^-^ > 1228 mg/l). Ratios of Na^+^ to Cl^-^ in these samples were above unity, suggesting that Na^+^ originated from the dissolution of silicate minerals (71). Previous studies have shown that surface thermal fluids in Kangding contain a volume percentage of CO_2_ greater than 95% (Guo et al., 2017). This compound favors the dissolution of silicate minerals, which are widely distributed in intrusive and metamorphic rocks in the ETPGB (58). If isochemical dissolution of albite had occurred, Na^+^ and HCO_3_^-^ would have been incorporated into the water in the same molar ratios as they were in the rock (72).

$$\mathrm{NaAlSi}_{3}O_{8}+\mathrm{CO}_{2}+{2H}_{2}O\leftrightarrow\mathrm{Na}^{+}+{Al(OH)}_{3}+\mathrm{HCO}_{3}^{-}$$

As seen in Figure 4B (i-ii), Kangding samples and Batang’s sample 1 located close to the equimolar line, but none of them adjusted completely. Since $H_{2}S$ may also have favored albite dissolution by producing Na^+^ and SO_4_^2-^ in 2:1 molar ratio, the joint action of CO_2_ and H_2_S was evaluated.

$$\mathrm{NaAlSi}_{3}O_{8}+H_{2}S+{2O}_{2}\leftrightarrow{2Na}^{+}+{6SiO}_{2}+2AlO\left( \mathrm{OH} \right)+\mathrm{SO}_{4}^{-}$$

Although H_2_S concentrations are low in the ETPGB (73, 74), the Kangding waters conformed better to the equimolar line with the addition of SO_4_^2-^ (Figure 4B (iii)). However, as Figure 4B (iv) illustrates, Kangding’s samples 2, 3, and 5 deviate from the equimolar line, which may be explained by analytical errors that could have been more evident at low concentrations of SO_4_^2-^. In contrast, ionic concentrations in the remaining Batang samples and sample 4 from Kangding did not appear to be dominated by albite dissolution.

Turning to the TVZ, Waikite bicarbonate waters have been interpreted as peripheral waters, being an outflow from the Waiotapu geothermal field (67, 75). In that case, bicarbonate may have originated from the interaction of CO_2_ with the surrounding rocks. This same origin is believed to have the bicarbonate-rich waters of Wairakei-Tauhara.
